# Decoding Wheat Endosphere–Rhizosphere Microbiomes in *Rhizoctonia solani*–Infested Soils Challenged by *Streptomyces* Biocontrol Agents

**DOI:** 10.3389/fpls.2019.01038

**Published:** 2019-08-26

**Authors:** Ricardo Araujo, Christopher Dunlap, Steve Barnett, Christopher M.M. Franco

**Affiliations:** ^1^Department of Medical Biotechnology, Flinders University, Adelaide, SA, Australia; ^2^i3S, University of Porto, Porto, Portugal; ^3^Crop Bioprotection Research, The United States Department of Agriculture, Peoria, IL, United States; ^4^South Australian Research & Development Institute (SARDI), Adelaide, SA, Australia

**Keywords:** 16S biodiversity, biocontrol agent, cereal microbiology, endophyte, ITS1 biodiversity, *Paenibacillus*, plant microbiome, *Streptomyces*

## Abstract

The endosphere and the rhizosphere are pertinent milieus with microbial communities that perturb the agronomic traits of crop plants through beneficial or detrimental interactions. In this study, we challenged these communities by adding *Streptomyces* biocontrol strains to wheat seeds in soils with severe *Rhizoctonia solani* infestation. Wheat plants were grown in a glasshouse standardized system, and the bacterial and fungal microbiomes of 233 samples of wheat roots (endosphere) and rhizosphere soils were monitored for 20 weeks, from seed to mature plant stage. The results showed highly dynamic and diverse microbial communities that changed over time, with *Sphingomonas* bacteria and *Aspergillus*, *Dipodascus*, and *Trichoderma* fungi increasing over time. Application of biocontrol *Streptomyces* strains promoted plant growth and maturation of wheat heads and modulated the root microbiome, decreasing *Paenibacillus* and increasing other bacterial and fungal OTUs. The soils with the highest levels of *R. solani* had increased reads of *Thanatephorus* (*Rhizoctonia* anamorph) and increased root disease levels and increased *Balneimonas*, *Massilia*, *Pseudomonas*, and unclassified *Micrococcaceae*. As we enter the era of biologically sustainable agriculture, it may be possible to reduce and limit the effects of serious fungal infestations by promoting a beneficial microbiome through the application of biocontrol agents during different periods of plant development.

## Introduction

Cereals represent the main carbohydrate food source in the world, particularly wheat that accounts for near 40% of the cereal supply worldwide (Charmet, 2011). For millennia, wheat has played a major role in the development of healthy societies and has supported economic and social stability (Würtenberger et al., 2006; Charmet, 2011). Intensive cereal cropping has shown an exponential increase in productivity and yield since the nineteenth century with the introduction of machinery and technology, but in the last few decades, the spread of soil infestations, soil degradation, and environmentally adverse conditions have been responsible for occasional decreases and instability in this cropping system (Lobell, 2009; Murray and Brennan, 2009). Understanding wheat crop system dynamics is critical, and several studies describe the rhizosphere as a pertinent milieu with microbial communities that perturb the agronomic traits through beneficial or detrimental interactions. The endosphere is the region inside the plant with microorganisms, namely, *Actinobacteria, Bacteroidetes*, and *Proteobacteria* (Mendes et al., 2013; Turner et al., 2013, Schlaeppi and Bulgarelli, 2015; Vandenkoornhuyse et al., 2015), which influence root health and plant growth. From a sustainable perspective, microbiome management is important to predict the profitability of agricultural production systems, avoid soil degradation, understand plant responses to environmental challenges, and identify which microbes are more sensitive to each cropping practice (Hirsch and Mauchline, 2012; Mendes et al., 2013; Massart et al., 2015; Schlaeppi and Bulgarelli, 2015; van der Heijden and Hartmann, 2016).

*Rhizoctonia* root rot caused by *Rhizoctonia solani* AG8 (Kühn, teleomorph *Thanatephorus cucumeris*) is a major root infestation of cereals and other crops in dryland cropping systems, causing stunted seedlings and resulting in reduced access to water and nutrients by the plant (Paulitz et al., 2003; Schillinger and Paulitz, 2006; Anees et al., 2010; Jaffar et al., 2016). This can result in areas of high infestation levels with noticeable reduction of plant growth, or “bare patches,” up to several square meters or up to 20% of the crop area (Schillinger and Paulitz, 2006; Anees et al., 2010). Infestation is increased in low rainfall areas resulting in low grain fill that exacerbates yield losses (Okubara et al., 2014; Sánchez-Cañizares et al., 2017). In Australia, *Rhizoctonia* infestation is most prevalent in the southern and western cropping regions, with registered and potential annual yield losses estimated at $59 million and $166 million, respectively (Murray and Brennan, 2009). *Rhizoctonia* root rot is difficult to control due to its wide host range (Cook et al., 2002), lack of commercially available resistant cereal cultivars, and increased prevalence in direct drill or minimal tillage practices (Schillinger and Paulitz, 2006). Current options for partial control include strategic tillage below seeds (Roget et al., 1996), removal of the green bridge with herbicide timing (Roget et al., 1987; Babiker et al., 2011), rotation with non-cereal crops (Angus et al., 2015), and in-furrow chemical fungicide treatments (Roget et al., 1987), and more recently, by using biocontrol-coated seeds (Franco et al., 2007; Barnett et al., 2019). Currently, the estimated cost of control measures is A$106 million annually (Murray and Brennan, 2009).

*Rhizoctonia* root rot can be influenced by root-associated microorganisms, and biocontrol agent–coated seeds represent a biologically sustainable alternative with increasing potential in agriculture (Barnett et al., 2006; Dua and Sindhu, 2012; Yin et al., 2013; Mavrodi et al., 2014; Barnett et al., 2017). Endophytic *Streptomyces* species have been tested for biocontrol of phytopathogens in broad-acre cropping systems because of their ability to produce secondary metabolites, including antibiotics, and induce systemic resistance in the plant (Franco et al., 2016; Conn et al., 2008; Barnawal et al., 2017). Biocontrol agents can enhance root and shoot lengths, plant weight, higher tiller numbers, and/or induction of early flowering (Yang et al., 2012; Bokati et al., 2016; Franco et al., 2016; Araujo et al., 2017; Franco et al., 2017; Wemheuer et al., 2017). In addition, these *Actinobacteria* produce spores for long-term viability and stability during storage (Emmert and Handelsman, 1999) and have the ability to produce siderophores (Wang et al., 2014), indole acetic acid (Khamna et al., 2009), and enzymes such as cellulases, chitinases, glucanases, and ACC deaminase (El-Tarabily, 2006; El-Tarabily, 2008). The enrichment of the root microbiome is a highly dynamic process that alters from the seed stage to the harvesting period. In order to understand and manage the microbiome, it is important to monitor the changes in microbial populations at each stage of plant growth (Turner et al., 2013; van der Heijden and Hartmann, 2016).

In the present study, we detail the dynamics of endosphere and rhizosphere microbiomes (both bacterial and fungal populations) in wheat plants for a period of 5 months. Wheat plants were grown in a standard glasshouse system in order to test the following hypotheses: 1) the endosphere and rhizosphere microbiomes of wheat crops change over time in a predictable manner, even in soils with severe *Rhizoctonia* infestation; 2) the addition of biocontrol *Streptomyces* strains (e.g., F11, EN16, or F5) impacts endophytic and rhizosphere microbial populations; and 3) specific microorganisms existing in the plant roots and rhizosphere soils respond to high levels of *Rhizoctonia* infestation, especially during the first weeks.

## Materials and Methods

### Biocontrol Cultures and Seed Coating

The strains F11, EN16, and F5 (all identified as *Streptomyces* sp.) (Franco et al., 2016) described as biocontrol agents (BCA) 1, 2, and 3, respectively, were used in this study. BCA1, BCA2, and BCA3 had previously reduced *Rhizoctonia* root rot in both pot bioassays and in the field and have demonstrated in vitro inhibition against *R. solani* (Barnett et al., 2017; Barnett et al., 2019). The strains were identified by 16S rRNA gene sequencing and stored in culture collections of endophytic bacteria kept at Flinders University. A concentrated suspension of each strain was prepared in 0.3% (w/v) xanthan gum sticker solution and applied to 20g wheat seeds to a final count of ≈10^5^ cfu/seed, as described in Barnett et al. (2017). Seeds were stored at room temperature for no more than 1 week before being used in pot bioassays. Seed cfu was assessed immediately and at 1, 2, and 7 d after application for confirmation of bacterial viability and concentration per seed (≈10^5^ cfu/seed) as in Barnett et al. (2017).

### Pot Bioassays in Glasshouse

Pot bioassays were prepared using field cropping soil collected at Waikerie, South Australia (34°14’32.91”S, 140° 5’44.31”E; details for soil features in **Supplement 1**). The bulk soil (150kg) was collected from the top 10cm of a 100-m^2^ section of the field, avoiding the collection of plants material larger than 2mm. This soil had a continuing *Rhizoctonia* problem with background levels of R. solani AG8 of 492 pg DNA/g soil, determined by PreDictaB™ (SARDI, Urrbrae, SA, Australia; http://www.pir.sa.gov.au/research/services/molecular_diagnostics/predicta_b), considered to carry a high risk of *Rhizoctonia* root rot (Ophel-Keller et al., 2008; Poole et al., 2015). There were no or low detectable levels of other root pathogens, such as *Pythium* sp., *Gaeumannomyces graminis* var. *tritici* (Ggt), *Fusarium pseudograminearum*, and *Fusarium culmorum*. Soil was air dried and sieved to <2 mm prior to use in pot bioassays. Soil chemical and physical soil properties were analyzed by CSBP Laboratories (https://www.csbp.com.au/CSBP-Lab, Perth, Western Australia, details in **Supplemental Material 1**). Water holding capacity was determined by the pressure plate method with a 1-m column (Marshall and Holmes, 1979) and the soil then adjusted to 60% water holding capacity for use. The pot experiments were prepared with the amount of soil per pot depending on the time of harvest: 600g for 4 weeks, 1,000g for 8 weeks, 1,125g for 12 weeks, 2,000g for 16 weeks, and 4,800g for 20 weeks. For half of the bioassays, three *R. solani* AG8 strain W19-infested millet seeds (https://www.keelangrainandfodder.com.au/) were placed in the center of each pot, for tests with higher levels of *R. solani* infestation, and the pots allowed to incubate for 1 week at 15°C in a controlled temperature room to allow for *R. solani* to colonize the soil. Then, five wheat seeds (*Triticum aestivum*), cultivar Yitpi, were planted per pot, covered with 50g of soil and 50g of coarse sand to reduce evaporation. Plants were grown in a 15°C room for the first 4 weeks and then were moved to a glasshouse under natural lightning and temperature conditions (mean temperature of 10 to 24°C during the autumn and early winter periods). The pots were watered twice a week to their original starting weight. Each pot used for testing time points, BCA treatments and soil infestation levels were run with four independent replicates arranged in a randomized complete block design. At 4, 8, 12, 16, and 20 weeks, plants were carefully washed and assessed for root rot disease on seminal and nodal roots using a 0–5 disease scale (0 = healthy roots with nodal and seminal roots, several secondary thin and long roots, no signs of disease; 5 = highly diseased and damaged nodal roots without seminal roots) (Roget, 1995). The number of plants per plot was also assessed at each time point. Pot bioassays were run from February to July 2016. Wheat plants were collected; the nodal and seminal roots were cut using sterilized scissors and washed at each time point to remove all the soil and organic matter; then, the surface of the roots was sterilized with sodium hypochlorite 2% (for 3 min) and ethanol 70% (for 3 min) and washed three times with sterile water. Rhizosphere soils were collected by recovering the small layer of soil on the surface of the roots; roots were initially collected, gently shaken to discard loosely adhering soil and the adjacent rhizosphere soil in the root surface collected by shaking the roots vigorously into a sterilized envelope (a sterile spatula was occasionally used on this procedure without damaging the roots—5g to 30g of rhizosphere soil was collected per independent pot/replicate).

### DNA Extraction, Sequencing, and Bioinformatics

Root and soil samples were randomized (random numbers were attributed to the packages before storing to blind sample processing), stored at −80°C and processed for DNA extraction (groups of 16 random samples were processed simultaneously without any specific order). A fixed amount of 5 seeds, 1g of seminal roots (nodal roots were used for 4-week roots with serious disease and less than 1g seminal root material), or 2g of rhizosphere soil was used per replicate and subjected to a CTAB DNA extraction strategy (Zhang et al., 2010). The final DNA obtained was suspended in TE buffer. Polymerase chain reaction (PCR) was performed using KAPA HiFi PCR master mix (KAPA Biosystems Willington, MA, USA) using the following parameters: 95°C, 10 min, and 35 cycles of 95°C, 30 s; 58°C, 30 s; and 72°C, 60s. PCR primers for the bacterial community targeted the V3–V4 regions of the 16S rRNA genes with 341F and 806R primers (Muyzer et al., 1993; Caporaso et al., 2011), while for the fungal community that targeted the ITS1 region was targeted with ITS1F and ITS2 primers (Gardes and Bruns, 1993). The primers were incorporated into fusion primers for Illumina dual indexing and incorporation of Illumina adapters (Caporaso et al., 2012). After PCR, the amplicons were cleaned and normalized using a SequalPrep^™^ normalization plate (Thermo Fisher Inc., Waltham, MA, USA). The samples were pooled and the library quantified with a KAPA Library Quantification Kit (KAPA Biosystems Willington, MA, USA). The samples were sequenced using an Illumina MiSeq System with a MiSeq V3 2 x 300 bp sequencing kit. QIIME 1.9 (Caporaso et al., 2010) and USEARCH 9.2.64 (Edgar and Flyvbjerg, 2015) workflows were used for read merging, chimera removal (uchime2), operational taxonomic unit (OTU) picking, and taxonomic assigning (Ribosomal Database Project v11.4). Sequences with ≥97% identity defined the OTUs following sequence alignment in accordance to the model organism priors Escherichia coli; the clustering was produced in two passes of the swarm algorithm v2.1.6 (the first pass with an aggregation distance equal to 1 and the second pass with an aggregation distance equal to 3). Amplicon sequence variants (ASVs) were identified using a previously suggested R pipeline and DADA2 method (Callahan et al., 2016); Greengenes database (gg_13_8_train_set_97) was used for the 16S rRNA amplicon classification and UNITE database (UNITE_public_28.06.2017) for the ITS amplicon classification. The cutoff of more than or equal to 10 reads was considered for OTUs and ASVs included in this study.

### Statistical Analysis

Plant and disease data from pot bioassays were analyzed as three-way factorial (five sampling times x two disease levels x four seed treatments) randomized complete block design with time fitted as a whole plot using GenStat version 14 (VSN International Ltd., Hemel Hempstead, England, UK). Fisher’s least significant difference (lsd) was used to compare treatment means as the data was near normally distributed with homogeneity of variance between factors; *Rhizoctonia*-disease severity was analyzed by Kendall’s coefficient of concordance (a non-parametric method). Data and statistical analyses were performed on Microsoft Office Excel 2013 (Microsoft Corporation, Santa Rosa, California, USA), STAMP 2.1.3 (Parks et al., 2014), PRIMER-6 (PRIMER-e, Auckland, New Zealand), and IBM SPSS Statistics 22 (IBM, New York, USA). Community diversity and distribution analyses were conducted by running analysis of similarities (ANOSIM) one-way analysis (calculating the resemblance and using similarity data type), non-metric multidimensional scaling (nMDS), clustering analysis (complete linkage), canonical analysis of principal components (CAP), homogeneity of dispersions (PermDISP; calculating the resemblance, similarity data type, using squared root of relative abundance (Legendre and Gallagher, 2001), Bray-Curtis similarities, and 999 permutations), and permutational multivariate analysis of variance (PERMANOVA) to reveal the effects of each factor (sample type, sampling time, biocontrol treatment or *Rhizoctonia* soil level) on the community composition (using squared root transformed data, Bray-Curtis similarities, and 4,999 permutations of residuals under a reduced model), and similarity percentages (SIMPER) analysis (using Bray-Curtis similarities and 90% cutoff for low contributions) (Anderson, 2001; Anderson et al., 2006; Clarke et al., 2008). Network analysis was conducted using the molecular ecological network analyses platform (http://ieg4.rccc.ou.edu/MENA/) (Deng et al., 2012) to generate the networks, Cytoscape (Shannon et al., 2003) to visualize it, and cytoHubba (Chin et al., 2014) using maximal clique centrality (MCC) scores to select the top taxonomic groups with links in roots and rhizosphere soil samples. Random matrix theory (RMT)–based modeling was used for network analysis as this approach is powerful in delineating phylogenetic molecular ecological networks in microbial communities (following some steps microbial sequence collection, data standardization, Pearson correlation estimation, adjacency matrix determination by an RMT-based approach, network characterization, and module detection) and building an adjacency matrix that represents interactions in a network graph (Zhou et al., 2011). The reads in each sample were converted into percentage values according to the total number of OTUs or ASVs in the sample to eliminate the effect of the final number of reads (Zaura et al., 2009). These values were then transformed using the Hellinger approach (Legendre and Gallagher, 2001) to reduce the effects of overestimation among the most common taxa and the values compared on dissimilarity matrices that could be used for multiple population analyses. Post hoc analyses were done for multiple groups using one-way analysis of variance (ANOVA), Tukey-Kramer (0.95), and Eta-squared for effect size, while two-group analysis used Welch’s t-test (two-sided, Welch’s inverted for confidence interval method). The other data were compared at a significance level of 0.05 by the ANOVA test using the Bonferroni correction and by Student’s t test (when the population could be assumed to be normally distributed) or Wilcoxon signed-rank test (when the population could not be assumed to be normally distributed) for paired samples.

## Results

### Microbial Diversity and the Effect of Biocontrol Strains in Wheat Plants

**Figure 1:**
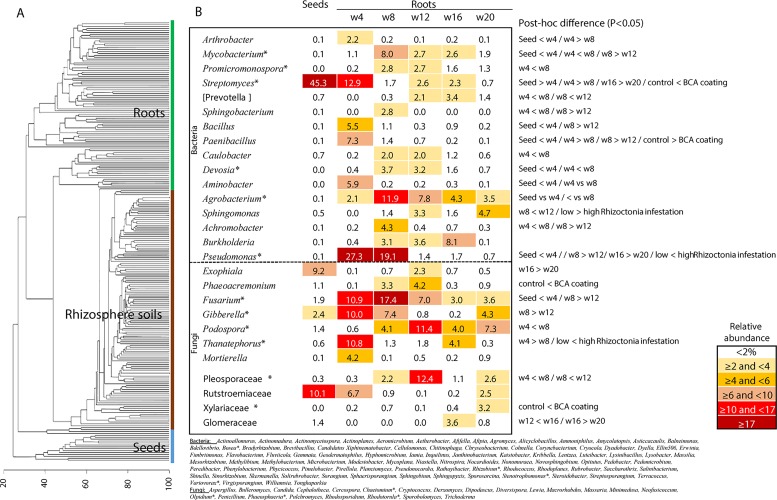
**(A)** Clustering analysis of all samples included in the study; **(B)** relative abundance of the core bacteria and fungi found in seeds and roots during the entire study (in the table the taxa with at least 2% in any sampling time; the complete list of core bacteria and fungi classified at genus level is below the table and includes the taxa with low relative abundance—* marks the taxonomic groups with core ASVs found in all sampling time points); the data for control and biocontrol treatments was pooled for these analyses. Details of *post hoc* plots can be seen in **Supplemental Materials 6**, **7**, and **10**.

Differences were observed on the wheat plants considering the studied factors: 1) *R. solani* level in the soil, 2) treatment with biocontrol strains (mainly F11 and EN16), and 3) sampling time. The agent F5 did not affect the plant growth, and the F5 plants were similar to the control wheat plants (in both soils with low and high *R. solani* levels). Wheat plants were obtained from control- and biocontrol-coated seeds grown in the glasshouse with the biocontrol-treated plants (F11 and EN16) having a higher biomass at later stages, earlier formation of wheat heads, and lower root disease indexes (more evident with EN16-coated seeds) (**Supplemental Material 2**). The roots and rhizosphere soils of each of these plants were then used for microbiome studies to compare untreated control versus biocontrol-treated plants (with F11, EN16, or F5 strains) in the presence of low and high levels of *R. solani* infestation. A total of 1,216,983 bacterial and 793,412 high-quality fungal sequences were organized into 6,880 bacterial and 861 fungal OTUs, or 16,248 bacterial and 969 fungal ASVs (details of ASVs in **Supplemental Material 3**). These sequences consisted of 628 bacterial and 204 fungal taxa (assigned at the genus or higher taxonomic levels) from the analyzed 233 samples. **Figure 1** shows the bacteria and fungi found in seed and root samples across the entire study (a set of 137 taxonomic groups were found in more than 75% of the samples, but only 13% of these taxa showed ASVs transversal to most of the collected samples); **Supplementary Material 4** shows the most frequent bacterial and fungal genera found in rhizosphere soils and wheat roots from the 20-week crop cycle. A set of 16 genera of bacteria and 7 fungi were found in all seed and root samples (**Figure 1**), being *Agrobacterium*, *Pseudomonas*, *Streptomyces*, and *Fusarium* the genera with the highest relative abundance in wheat roots or seeds.

The Shannon diversity indices for microbial communities of root and rhizosphere are shown in **Table 1**. This index was systematically higher in the rhizosphere soils compared to root or seed samples. The diversity was slightly decreased from the initial seeds or soils to 4-week sampled roots or rhizosphere soils, but then the diversity index increased in the following weeks. **Table 2** shows the statistical differences found in the microbial populations considering the multiple factors; ANOSIM showed a clear distinction between seeds, roots, and rhizosphere soil samples (sample types). Then, it also revealed the sampling time as the strongest factor (P < 0.001) responsible for the richness and composition of the microbial communities found in roots and rhizosphere samples, in comparison with the other factors biocontrol treatments and *Rhizoctonia* soil levels (**Tables 2** and **3**). *Streptomyces* biocontrol agents tested in this study showed a significant effect on the root microbial populations resulting in distinct microbiomes in the endosphere and rhizosphere (**Table 3** and **Supplemental Materials 5** and **6**); the effect of *R. solani* levels on root and rhizosphere microbiome was low and only significantly different by ANOSIM analysis for the root microbiome (**Table 3**). High levels of *R. solani* showed a significant alteration of the rhizosphere soil communities from 4 to 12 weeks, not in the subsequent weeks, suggesting some rhizosphere microorganism may respond to root disease.

**Table 1:** Shannon diversity index and Margalef richness (at genus or higher taxonomic level) for wheat root and rhizosphere soil samples; average (minimum and maximum values).

		Shannon diversity	Shannon diversity	Margalef richness	Margalef richness
		Root	Rhizosphere	Root	Rhizosphere
	All	4.25 (*2.32–6.02)*	5.52 *(4.68–6.04)*	86	223
Sampling	Seed stage	3.41 (2.64–4.31)*	5.79 (5.08–6.25)*	31*	345*
time (weeks)	4	3.29 *(2.32–4.14)*	5.39 *(4.72–5.66)*	26	188
	8	4.24 *(3.49–4.71)*	5.49 *(5.28–5.77)*	76	221
	12	4.73 *(4.01–5.43)*	5.51 *(4.68–5.75)*	132	230
	16	4.50 *(3.21–6.02)*	5.67 *(5.44–6.04)*	93	252
	20	4.82 *(3.74–5.53)*	5.51 *(5.12–5.88)*	140	219
Biocontrol	Control	4.26 *(3.02–5.53)*	5.54 *(4.72–6.04)*	82	227
treatment	F11	4.24 *(2.32–5.37)*	5.51 *(4.68–5.76)*	86	223
	EN16	4.36 *(2.66–6.02)*	5.51 *(5.17–5.88)*	93	215
	F5	4.15 *(3.15–5.16)*	5.53 *(5.12–6.00)*	83	228
Rhizoctonia	Low level	4.38 *(2.99–5.28)*	5.59 *(4.68–6.04)*	97	223
soil level	High level	4.10 *(2.32–6.02)*	5.54 *(5.12–5.76)*	73	227

*Values for seeds (root column) or initial soil (rhizosphere column).

**Table 2:** P values for analysis of similarities (ANOSIM) in roots and rhizosphere soil samples. The results represent the same samples according to sampling time, the biocontrol treatment, and finally according to pathogen infection level groupings.

	Roots	Rhizosphere soils
Sample	**0.001**	**0.001**
Sampling time	**0.001**	**0.001**
Biocontrol treatment	**0.044**	**0.714**
*Rhizoctonia* level	**0.009**	**0.319**

Bold mean the P value is significant (P<0.05). Analyses done in Primer v6 using squared root transformation of the data and resemblance Bray–Curtis similarity (with dummy variable).

**Table 3:** P values for analysis of similarities (ANOSIM) for each group of samples (values per week considering the effect of biocontrol agents and different levels of *Rhizoctonia* disease).

			ANOSIM
**Roots**	Week 4	Biocontrols	**0.001**
		*Rhizoctonia*	0.111
	Week 8	Biocontrols	**0.003**
		*Rhizoctonia*	0.816
	Week 12	Biocontrols	**0.032**
		*Rhizoctonia*	0.682
	Week 16	Biocontrols	0.671
		*Rhizoctonia*	0.574
	Week 20	Biocontrols	0.141
		*Rhizoctonia*	0.5
**Rhizosphere**	Week 4	Biocontrols	0.451
**soils**		*Rhizoctonia*	**0.004**
	Week 8	Biocontrols	0.6
		*Rhizoctonia*	**0.012**
	Week 12	Biocontrols	0.61
		*Rhizoctonia*	**0.038**
	Week 16	Biocontrols	0.388
		*Rhizoctonia*	0.363
	Week 20	Biocontrols	0.441
		*Rhizoctonia*	0.853

Bold mean the P value is significant (P<0.05).

### Endosphere and Rhizosphere Microbiomes Over a 5-Month Period

**Figure 2:**
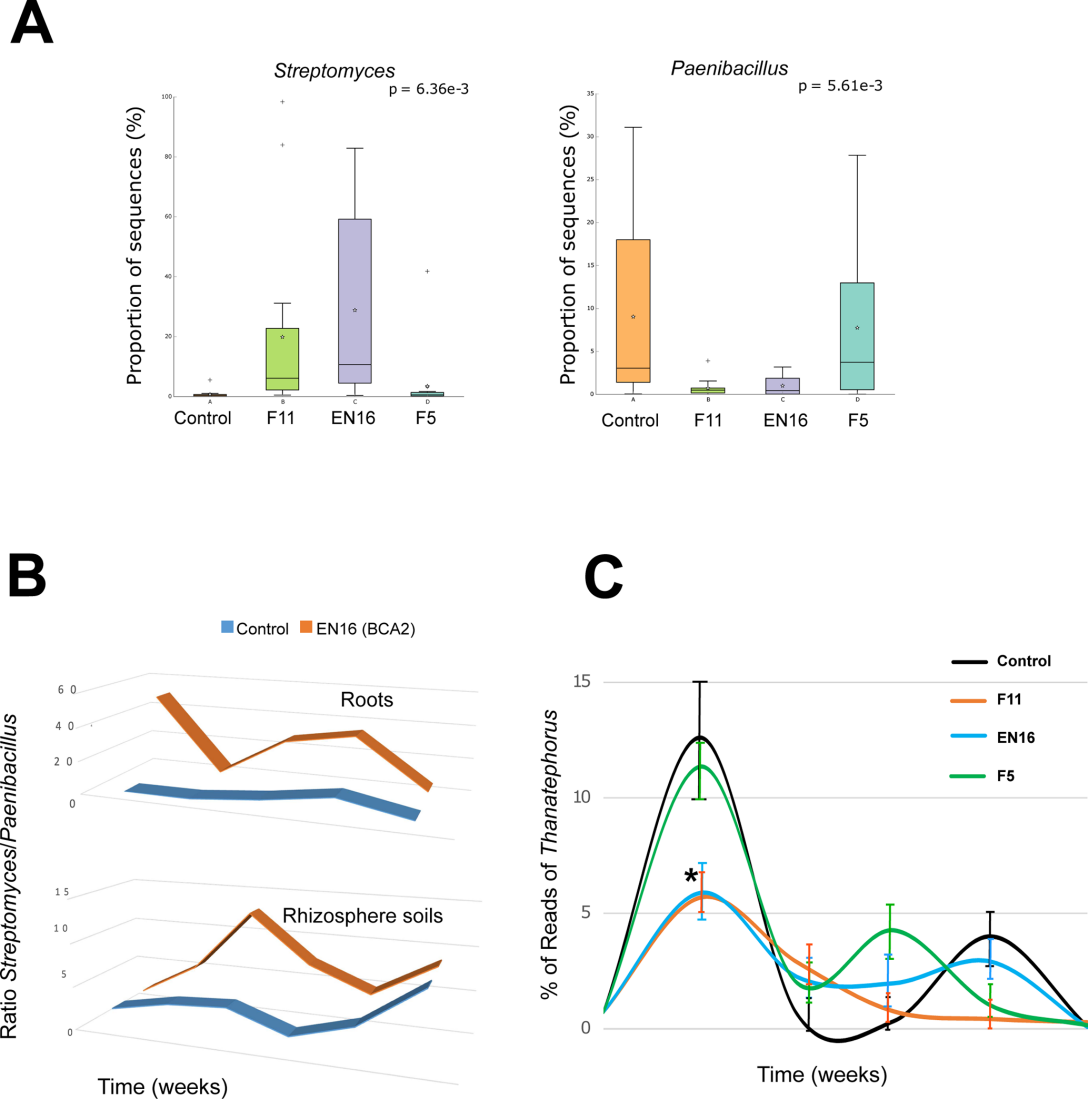
Percentage **(A)** and ratio **(B)** and of *Streptomyces* and *Paenibacillus* sequences in roots and rhizosphere soils; relative abundance of *Thanatephorus* reads (within fungal population) ± SEM in root samples (*P < 0.05) **(C)**.

A succession of microorganisms was observed in the wheat roots from 4 weeks to the mature plant after 20 weeks (**Figure 1** and **Supplemental Material 3**), with bacterial biodiversity being more prominent in the initial stages and fungal biodiversity increasing after the 12^th^ week. Some bacterial and fungal OTUs were maintained in the plant root for several weeks, while others were only identified occasionally (**Figure 1** and **Supplemental Materials 3** and **4**). Although *Streptomyces* and *Paenibacillus* were predominantly found in the roots at week 4 (**Figure 1** and **Supplemental Material 3**), the OTUs of these bacterial genera were not mainly found in the same set of roots and rhizosphere samples. *Streptomyces* dominated samples obtained from F11- and EN16-treated plants, while Paenibacillus were abundant on control and F5-treated plants (**Figures 2A, B** and **Supplemental Material 5**). Similarity percentage (SIMPER) analyses also showed an abundance of *Arthrobacter*, *Bacillus*, and *Paenibacillus* OTUs in the control roots soon after 4 weeks. Other OTUs increased in F11- and EN16-treated roots, mainly fungal OTUs, were classified as *Exophiala*, *Phaeoacremonium*, and unclassified *Xylariaceae* (see post hoc plots in **Supplement 6**). The comparison of the OTUs in the control roots versus the biocontrol-treated roots showed a similarity in less than 10% at single time points (specific weeks), increasing to nearly 20% when the total 5-month period was considered (data not shown). By comparing the taxonomic groups (at genus level) of the OTUs for the control versus biocontrol-treated roots at the same time point (week), the similarity ranged from 43 to 88% (**Table 4**); the similarity was maximum at the 8^th^ week of the wheat growth cycle for both bacteria and fungi found in roots. The relative abundance of the major and most common taxa found on wheat roots also showed differences over time (**Figure 1** and **Supplemental Information 7**). Among the rhizosphere soil samples, the taxonomic similarity was consistent over time (around 70% in all samples), and multiple ASVs were found in common over the weeks (**Supplement 3**).

**Table 4:** Taxonomic similarity (%) comparing control versus biocontrol-treated roots; taxonomic groups observed in roots obtained from low and high *Rhizoctonia*-level soils were also compared.

	Control vs. F11	Control vs. EN16	Control vs. F5	Low vs. high *Rhizoctonia*	Shannon diversity within the samples

4 weeks	63	64	48	43	Increase of bacterial and decrease of fungal biodiversity compared to seeds (*P* < 0.05)
8 weeks	88	88	88	88	Large increase of bacterial biodiversity (*P* < 0.05)
12 weeks	59	54	69	53	Large increase of fungal biodiversity (*P* < 0.05)
16 weeks	71	44	69	51	Slight decrease of bacterial and fungal biodiversity (not significant *P* > 0.05)
20 weeks	67	46	60	36	Slight increase of bacterial and fungal biodiversity (not significant *P* > 0.05)

**Figure 3:**
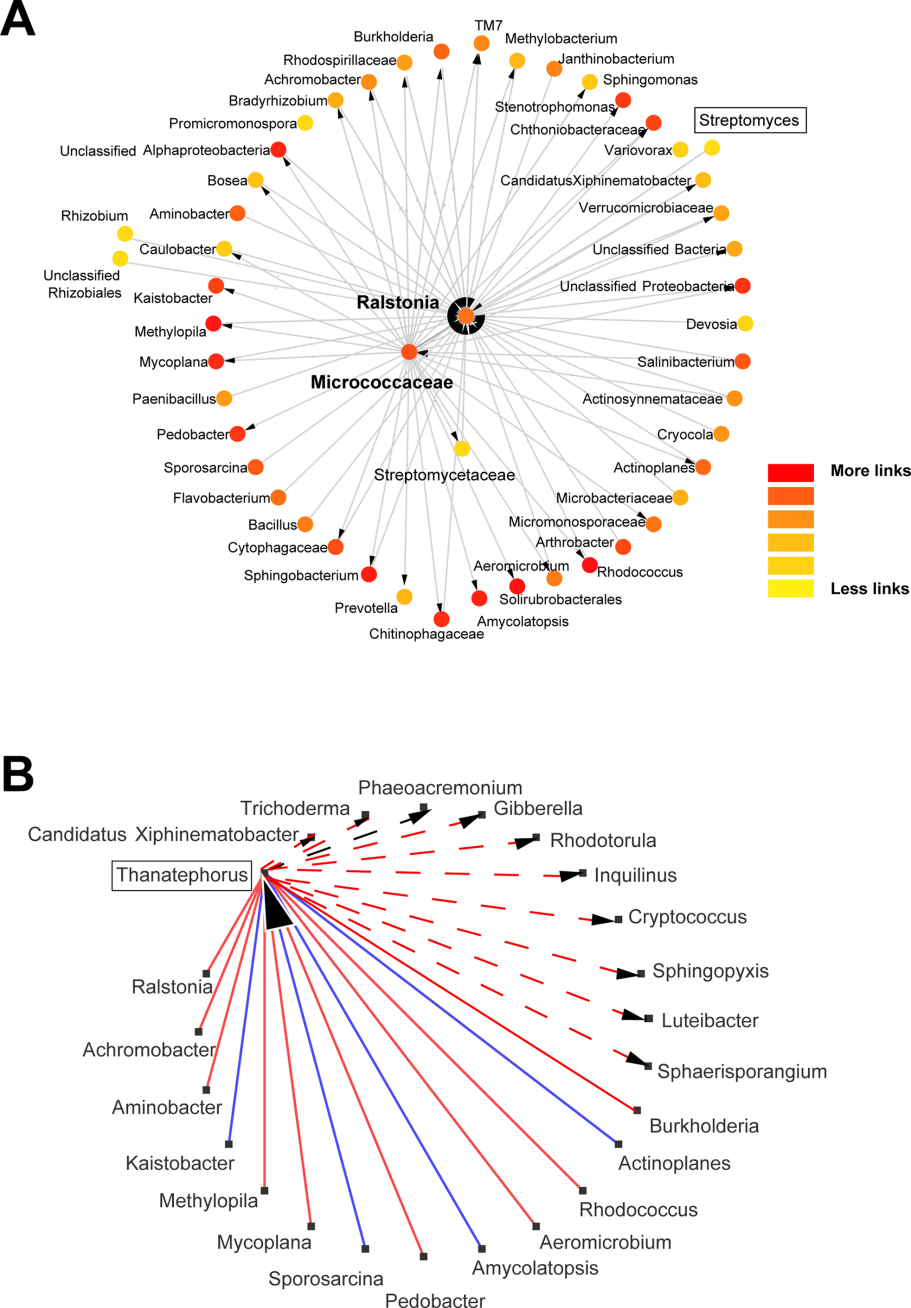
Network analysis for bacterial and fungal groups found in the wheat roots. Detailed networks were selected for *Streptomyces* and its neighbors **(A)**, and *Thanatephorus* connections **(B)** to visualize which taxonomic groups are directly affected by these taxa; negative and positive interactions are marked red and blue, respectively. Network analyses were conducted using molecular ecological network analysis pipeline (MENA; http://ieg4.rccc.ou.edu/MENA/) to generate the networks with a cutoff of 0.9 and Cytoscape environment to visualize and redesign the networks. Yellow to red color in network A means more links within the network.

Distinct dynamics were found among bacterial and fungal OTUs: 1) *Pseudomonas* OTUs were high in the 4- and 8-week sampled roots and reduced after the 12^th^ week (**Figure 1** and **Supplemental Information 7**); 2) *Sphingomonas* OTUs were particularly high after the 12^th^ and 20^th^ weeks (**Figure 1**); 3) Podospora OTUs increased after the 8^th^ week (**Figure 1** and **Supplemental Information 7**); 4) *Bacillus*, *Curvularia*, and Rubrobacter OTUs reduced over time in the rhizosphere soils (**Supplemental Material 8**); 5) *Devosia* was abundant at 8^th^ and 12^th^ weeks (**Figure 1**); and 6) *Aspergillus*, *Dipodascus*, *Rhodoplanes*, and *Trichoderma* OTUs (P < 0.05 using post hoc two-group analysis) were more abundant in later periods (**Supplemental Material 8**). Notably, molecular ecological network analyses showed Streptomyces as a lateral genus in the population analyses, directly interacting with Ralstonia, and barely interacting with other groups (**Figure 3A**); *Afifella*, *Luteibacter*, *Methylibium*, and *Shinella* were found in the center of the bacterial network analyses with the major number of links within the microbiome (**Supplemental Material 9**). Additionally, *Thanatephorus* (anamorph of *Rhizoctonia*) was found in the core of the network interacting with multiple bacteria and fungi (see **Figure 3B** for details).

### Biocontrol Strains and Other Taxa in Rhizoctonia Conducive Soils

**Figure 2C** shows the differences observed in reads of *Thanatephorus* OTUs (anamorph of *Rhizoctonia*) during the study. The highest values were seen at 4 weeks followed by a decrease of *Thanatephorus* OTUs in roots and rhizosphere soils in subsequent weeks. These values correlated with the disease incidence rate measured in the plant roots (**Supplemental Material 2**) showing higher root disease index at 4 weeks and decreasing in the weeks after. The relative abundance of *Thanatephorus* reads detected in F11- and EN16-treated roots at 4 weeks was significantly lower (P < 0.05) compared with the values for control roots; in the remaining sampling times, the relative abundance of *Thanatephorus* reads was less than 4%, and the differences were not significant (P > 0.05). Besides this direct effect observed by *Streptomyces* on the roots of plants exposed to *R. solani*–induced infestation, SIMPER, and ANOVA (STAMP) comparative analyses agreed that some other OTUs, classified as *Balneimonas* (*Bradyrhizobiaceae*), *Massilia* *(Oxalobacteraceae)*, *Pseudomonas,* and unclassified *Micrococcaceae*, *Rhizophlyctidaceae*, and *Gemmatimonadaceae* were particularly dominant in the soils with highest levels of *R. solani* (**Supplemental Materials 10** and **11**). OTUs of *Bradyrhizobiaceae* (e.g., *Balneimonas*) and *Micrococcaceae* were mainly present in the rhizosphere soils, while some OTUs of *Pseudomonas* increased with higher levels of root disease.

## Discussion

The microbiome of root endosphere and rhizosphere soils was monitored through the growth cycle (5 months) of the wheat crop growing in *Rhizoctonia*-infested soils with and without the influence of *Streptomyces* biocontrol strains tested at each step of plant development. This study confirmed that *Streptomyces* isolates (F11 and EN16) could modulate endosphere and rhizosphere microbiomes resulting in increased plant growth, reduced root disease, and increased number of wheat heads over the weeks. The third strain, *Streptomyces* F5, was less effective on plant physiology and produced a distinctively different microbiome compared with the first two endophytes. The addition of Streptomyces strains F11 and EN16 affected mostly *Paenibacillus* populations, commonly found in seeds (Yang et al., 2017), reducing their relative abundance drastically over the first weeks. In addition, the abundance of the OTUs of *Streptomyces* and the fungal OTUs classified as *Exophiala* (also found in seeds), *Phaeoacremonium*, and unclassified *Xylariaceae* increased during the same period. It is known that the enrichment of endosphere and rhizosphere microbiomes benefits the wheat plants (Yang et al., 2012; Bulgarelli et al., 2013; Reinhold-Hurek et al., 2015), and we have demonstrated that individual strains can shift the microbiome (not affecting the richness or evenness) and benefit the plants. It is possible that the efficacy of *Streptomyces* strains reported in glasshouse systems against *Rhizoctonia* in wheat (Goudjal et al., 2013) and tomato (Sabaratnam and Traquair, 2002) can produce predictable changes in the endosphere microbiome. The inoculum concentration of ≈10^5^ cfu/seed has shown benefits for wheat plants in field trials (Barnett et al., 2017). A higher inoculum in the initial seeds might result in 3–7% rise of the endophyte population in wheat roots (Conn and Franco, 2004). Increasing numbers of reports suggest that a high biodiversity at the endosphere and rhizosphere levels may give extra protective “tools” to plants to respond to environmental constraints and infestation stresses (Ratnadass et al., 2012; Reinhold-Hurek et al., 2015; van der Heijden and Hartmann, 2016; Barnawal et al., 2017).

It is now known that the OTUs and taxonomic groups detected at each stage of wheat growth change over time (Lauber et al., 2013; Reinhold-Hurek et al., 2015; Rascovan et al., 2016; Mahoney et al., 2017). Such patterns were also observed in this study with *R. solani*–infested soils, independently of the disease levels. Bacterial OTUs tend to be dominant in the initial stages of plant growth, colonize the wheat roots, and reach successive peaks of biodiversity during the first 12 weeks. While some bacterial OTUs are dominant during the initial weeks, other bacteria, such as *Sphingomonas*, were found mostly in the later stages of wheat root maturation. *Sphingomonas* are usually found in multiple parts (roots, leafs, flowers) of mature plants and also have a plant protective role against infestations (Kim et al., 1998; Khan et al., 2014). These bacteria are common plant endophytes and are known to benefit plants by producing phytohormones and support plant maturation processes (Khan et al., 2014; Asaf et al., 2017). Later, fungi gain more relevance and intensively colonize wheat roots; from the 12^th^ to the 16^th^ week, fungal biodiversity increased greatly, and some genera, such as *Aspergillus*, *Dipodascus*, and *Trichoderma*, described as protective for wheat and other plants (Nicolaisen et al., 2014; Bokati et al., 2016; Hertz et al., 2016; Barnett et al., 2017; Liu et al., 2018) were particularly abundant. In fact, it was clear that the microbial population changed over time, and distinct microbiomes can actually be considered for each stage of the wheat crop (**Supplement 3**) with only a fraction of OTUs/ASVs persisting during the entire period of this study (**Figure 1**).

Microbial communities were primarily affected by the sampling time of wheat roots, similar to findings for *Arabidopsis thaliana* (Bulgarelli et al., 2012), but at a second level, it was possible to observe some differences in the endosphere and rhizosphere microbiomes in the presence of effective biocontrol agents. Notably, *Streptomyces* did not show the highest degree of connectivity in the network analyses, being the positions with more interactions taken by other taxonomic groups such as *Afifella*, *Luteibacter*, *Methylibium*, *Shinella*, and *Chitinophaga*, that are frequent colonizers of the rhizosphere and previously described as relevant endophytes for nuts, sugarcane, or potato plants (Manter et al., 2010; Chakraborty et al., 2016; Taulé et al., 2016; Su et al., 2017; Lo et al., 2018). These may represent keystone microbes in the rhizosphere microbiome (Berry and Widder, 2014). These bacteria networked to several others certainly represent interesting targets for microbial interaction studies, but further analysis of the soils with increased levels of *R. solani* showed similar or lower relative abundance of these bacteria compared with the soils with low levels of *Rhizoctonia*. Instead, a higher contribution of other lateral taxonomic groups (*Streptomyces* or *Paenibacillus*) was found to interfere with the wheat root endosphere communities from *Rhizoctonia*-infested soils.

Soils with low and high levels of *R. solani* were challenged by biocontrol strains and its effects on endosphere and rhizosphere microbiomes compared. The number of reads of *Thanatephorus* (anamorph of *R. solani*) was not constant throughout the study, being peaks of reads collected in the initial weeks changing similarly to the values of disease severity seen in the wheat roots—higher values (roots were rated 4 and 5) in the first weeks and then decreasing to much lower levels (roots were rated 1 and 2) after the 12^th^ week. The interaction between *R. solani* within the endosphere and rhizosphere microbiomes was complex with interactions between multiple taxonomic groups, some of these representing well-known endophytes or plant benefiting microbes (e.g., *Trichoderma*, *Gibberella*, and *Burkholderia*) with mechanisms of action against soil infestations (Panea et al., 2013; Gouda et al., 2016) and others being central taxa in the network analyses (e.g., *Luteibacter*). Nevertheless, the presence of F11 and EN16 strains on roots resulted in lower number of reads of *Thanatephorus* at 4 weeks, in agreement with lower disease severity reported in the plants. This suggests the percentage of each OTUs in the microbiome profiles may provide a semi-quantitative perception of the infestation levels in the soils. Besides the impact of biocontrol *Streptomyces* on *Rhizoctonia* root rot, some *Pseudomonas* OTUs in wheat roots and OTUs of *Bradyrhizobiaceae*, *Micrococcaceae*, and *Oxalobacteraceae* (e.g., *Massilia*) in rhizosphere soils were positively responsive to the higher levels of *R. solani* inoculation. Some strains of these groups may also play a protective role against this specific infestation, and occasional isolates of *Massilia* were described with such properties (Yin et al., 2013). Antifungal activity was reported for multiple members of *Oxalobacteraceae*, including *Collimonas* spp., against the ectomycorrhizal fungus *Laccaria bicolor*, also affecting the growth and hyphae branching of the fungus and potentially modulating the fungal gene in stress response (Leveau et al., 2010). It remains to be clarified if the “manipulation” of all these taxonomic groups simultaneously in standardized experiments can in fact result in a much higher physiological effect in wheat plants than the ones we observed with *Streptomyces* strains alone. The proliferation of these microbes at the right stage of the wheat crop, both in the plant and rhizosphere soil, may be a highly efficient barrier against the spread of fungal infestations, keeping the plant and the biodiversity of the soils stable. The addition of selective treatments to seeds, either as inoculants or materials to increase specific taxa, may add even more productivity to crops in coming years (Li et al., 2018), being important to integrate and complement these approaches.

In conclusion, the microbial populations of both endosphere and rhizosphere soils experience major changes from the early stages to the flowering phase with distinct groups of microbes dominating each stage. The addition of effective biocontrol *Streptomyces* strains impact the microbiome as these strains take over the dominant place of other bacteria, e.g., *Paenibacillus*, in the wheat root. *Paenibacillus* had higher relative abundance within the endophytic communities of some fruits, such as apple (Liu et al., 2018), suggesting that some observations of this study may be extended to these plants. Disease variation levels in the soil may be monitored by routine comparison of the endosphere microbiome profiles, which may also reveal OTUs directly responding to major levels of *R. solani* in the soils. We are entering the era of biological sustainability strategies for consolidation and promotion of plant productivity by acting at multiple levels to reduce and limit the consequences of serious infestations. By promoting, monitoring, and controlling the microbiome and the biocontrol agents within the plant at each period of development, we may effectively achieve exceptional nutritional and environmental standards.

## Contribution to the Field Statement

*Rhizoctonia* root rot is a major root infestation affecting cereals and other crops that cases considerable damage to the plant reducing access to water and nutrients. The estimated cost of control measures is of several hundreds of million dollars annually in Australia, being the options for partial control restricted to tillage, use of herbicides and other chemicals and rotation with non-cereal crops. The use of biocontrol strains, mainly *Streptomyces*, as seed coats promote wheat growth and plant maturation resulting in changes in the endosphere and rhizosphere soil microbiomes. These changes may impact Rhizoctonia infestation at root level and limit the damages. In this study, specific bacterial and fungal OTUs responded to crop age, addition of biocontrol strains (the effects of three strains were compared) and increased levels of *Rhizoctonia solani* infestation in the soils. Some OTUs of *Balneimonas, Massilia*, *Pseudomonas* and unclassified Micrococcaceae responded essentially in the soils representing potential protectors against *Rhizoctonia* infestation advance, without damaging the soils or affecting its bacterial and fungal biodiversity. The application of biologically sustainable approaches in agriculture may limit the damaging effects of serious fungal infestations and preserve high levels of microbial biodiversity in the soils.

## Data Availability

The dataset supporting the conclusions of this article is available in the NCBI repository, under the ID SUB4129508, biosample SUB4129509 and bioproject PRJNA471385 (SRA study SRP149964; accessible with the link ftp://ftp-trace.ncbi.nlm.nih.gov/sra/review/SRP149964_20190318_110809_27e795eb0f314edf0479737480ab0f2a).

## Author Contributions

All authors participated in the design, conception and implementation of the study, data analyses, manuscript writing and elaboration.

## Funding

RA was supported by an Endeavour Postdoctoral Fellowship. This study was financed by Grains Research and Development Corporation (GRDC) project no. UF00008.

## Disclaimer

The biocontrol agents included in this study are protected under the Australian Provisional Application No. 2017901523, filed April 27, 2017, and U.S. Provisional Application No. 62/568,763, filed October 5, 2017. The strains are stored in Flinders University and the South Australian Research and Development Institute (SARDI) and are available for research studies. Mention of trade names or commercial products in this publication is solely for the purpose of providing specific information and does not imply recommendation or endorsement by the U.S. Department of Agriculture. USDA is an equal opportunity provider and employer.

## Conflict of Interest Statement

The authors declare that the research was conducted in the absence of any commercial or financial relationships that could be construed as a potential conflict of interest.
