# Supplementary material for: Decoding Wheat Endosphere–Rhizosphere Microbiomes in Rhizoctonia solani–Infested Soils Challenged by Streptomyces Biocontrol Agents
Source: Front Plant Sci. 2019 Aug 26;10:1038. doi: 10.3389/fpls.2019.01038 (PMC6718142; doi:10.3389/fpls.2019.01038)
Supplement: Supplementary file 1 [file DataSheet_1.zip › Data Sheet 1/Supplement1.pdf]

Supplemental material 1. Waikerie soil features used in the study.

		Waikerie
Depth		0-10
Colour		LTBR
Gravel	%	0
Texture		2.0
Ammonium Nitrogen	mg/Kg	1
Nitrate Nitrogen	mg/Kg	9
Phosphorus Colwell	mg/Kg	27
Potassium Colwell	mg/Kg	229
Sulphur	mg/Kg	2.8
Organic Carbon	%	0.60
Conductivity	dS/m	0.092
pH Level (CaCl2)	pH	6.9
pH Level (H2O)	pH	7.8
DTPA Copper	mg/Kg	0.23
DTPA Iron	mg/Kg	4.22
DTPA Manganese	mg/Kg	1.85
DTPA Zinc	mg/Kg	2.97
Exc. Aluminium	meq/100g	0.014
Exc. Calcium	meq/100g	5.14
Exc. Magnesium	meq/100g	0.75
Exc. Potassium	meq/100g	0.59
Exc. Sodium	meq/100g	0.04
Aluminium CaCl2	mg/Kg	< 0.20
Boron Hot CaCl2	mg/Kg	0.55
