# Supplementary material for: Decoding Wheat Endosphere–Rhizosphere Microbiomes in Rhizoctonia solani–Infested Soils Challenged by Streptomyces Biocontrol Agents
Source: Front Plant Sci. 2019 Aug 26;10:1038. doi: 10.3389/fpls.2019.01038 (PMC6718142; doi:10.3389/fpls.2019.01038)
Supplement: Supplementary file 1 [file DataSheet_1.zip › Data Sheet 1/Supplement2.pdf]

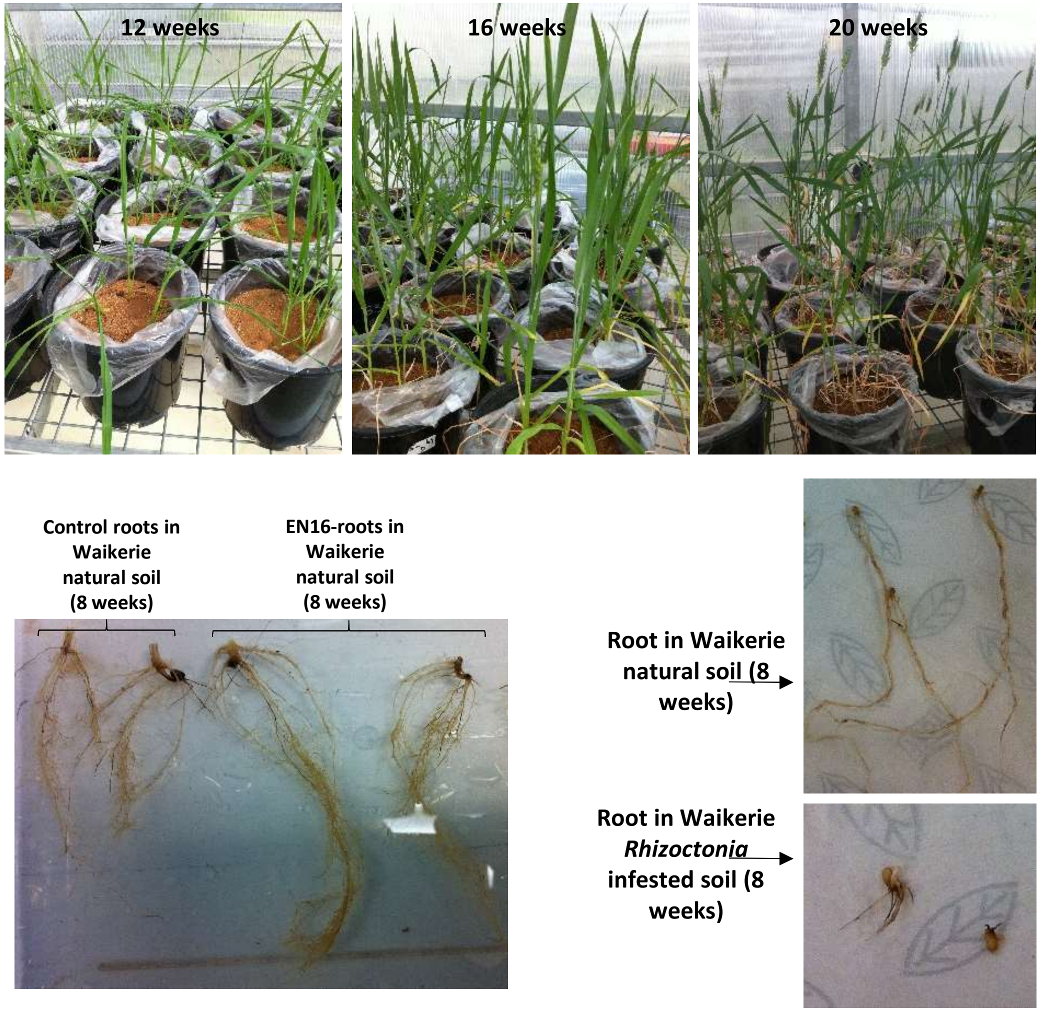

Table S1	*Rhizoctonia*-disease severity
Waikerie soil + control seed	2.4 ± 0.79
Waikerie soil + seed with F11	2.7 ± 0.75
Waikerie soil + seed with EN16	1.9 ± 0.62
Waikerie soil + seed with F5	2.4 ± 0.52
Waikerie *R. solani* infested soil + control seed	4.4 ± 0.48
Waikerie *R. solani* infested soil + seed with F11	4.7 ± 0.11
Waikerie *R. solani* infested soil + seed with EN16	3.9 ± 0.10
Waikerie *R. solani* infested soil +seed with F5	4.7 ± 0.83

Table S2	Weight per plant (mg) at 16 weeks
Waikerie soil + control seed	483 ± 51.0
Waikerie soil + seed with F11	602 ± 158.9
Waikerie soil + seed with EN16	569 ± 51.4
Waikerie soil + seed with F5	615 ± 138.7
Waikerie *R. solani* infested soil + control seed	571 ± 146.9
Waikerie *R. solani* infested soil + seed with F11	560 ± 150.3
Waikerie *R. solani* infested soil + seed with EN16	540 ± 123.6
Waikerie *R. solani* infested soil +seed with F5	558 ± 34.4

Table S3	Wheat heads at 20 weeks (average per pot)
Waikerie soil + control seed	4.5 ± 0.5
Waikerie soil + seed with F11	4.0 ± 0.4
Waikerie soil + seed with EN16	5.3 ± 0.9
Waikerie soil + seed with F5	4.0 ± 0.7
Waikerie *R. solani* infested soil + control seed	3.3 ± 0.3
Waikerie *R. solani* infested soil + seed with F11	4.3 ± 0.5
Waikerie *R. solani* infested soil + seed with EN16	4.75 ± 0.9
Waikerie *R. solani* infested soil +seed with F5	3.5 ± 0.9

Supplemental information 2. Plants grown in the glasshouse in pots at 12, 16 and 20 weeks, roots of control and EN16-treated plants at 8 weeks and comparison of roots in the native and *Rhizoctonia* high-level soils at 8 weeks. Tables S1, S2 and S3 show the values of *Rhizoctonia* disease severity at 4 weeks, weight per plant at 16 weeks and wheat heads per pot at 20 weeks, respectively - significant differences ($P<0.05$) are marked in colour.
