# Supplementary material for: Decoding Wheat Endosphere–Rhizosphere Microbiomes in Rhizoctonia solani–Infested Soils Challenged by Streptomyces Biocontrol Agents
Source: Front Plant Sci. 2019 Aug 26;10:1038. doi: 10.3389/fpls.2019.01038 (PMC6718142; doi:10.3389/fpls.2019.01038)
Supplement: Supplementary file 1 [file DataSheet_1.zip › Data Sheet 1/Supplement3.pdf]

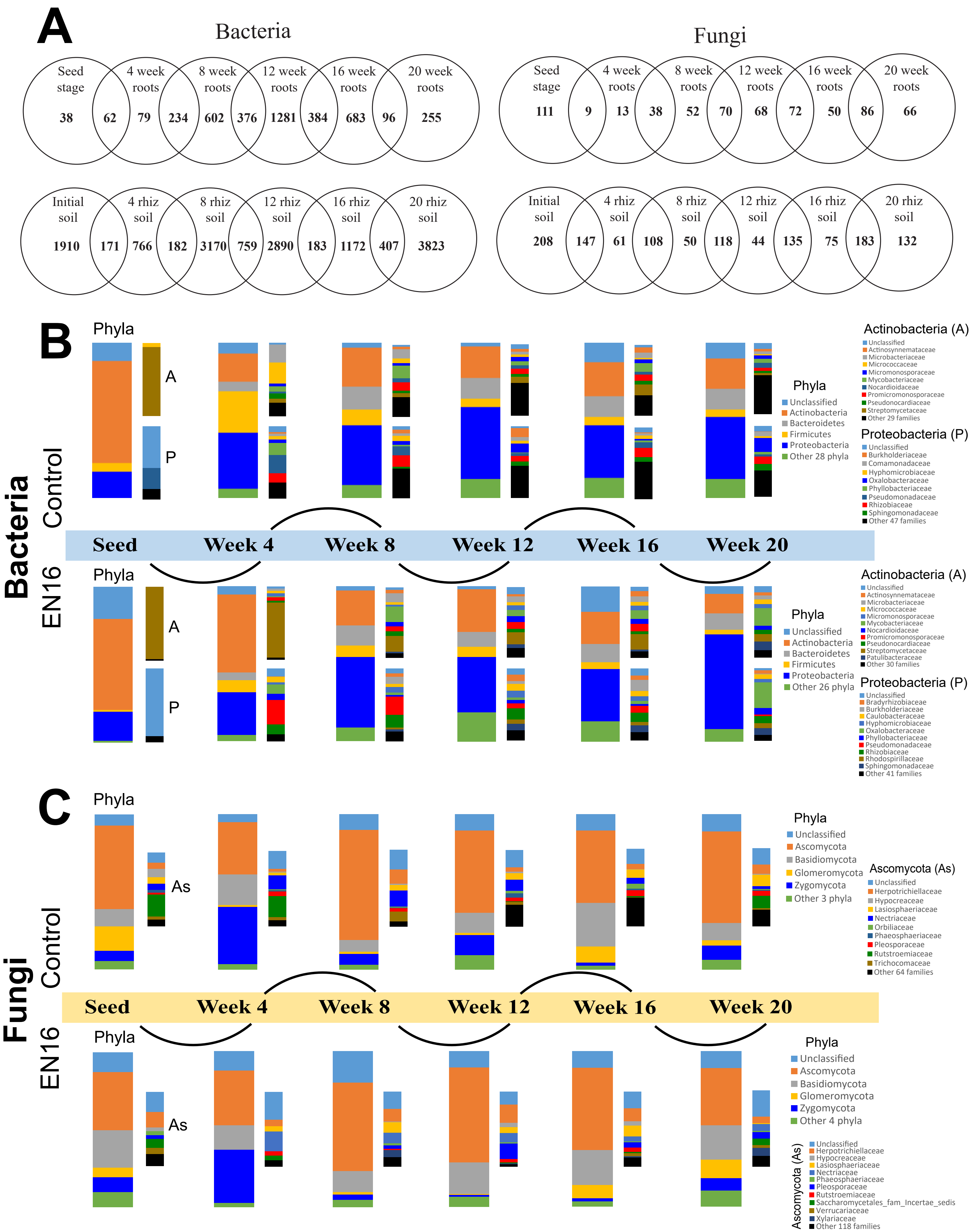

Supplemental material 3. A) Amplicon sequence variants (ASV) unique and common along the sampling weeks in seeds/roots and initial/rhizosphere soils. Relative abundance of Bacterial (B) and fungal (C) families in wheat seed and root samples along 20 weeks period comparing control versus EN16-treated samples. Specific profiles for Actinobacteria (Ac), Proteobacteria (Pr) and Ascomycota (As) are shown.
