# Supplementary material for: Decoding Wheat Endosphere–Rhizosphere Microbiomes in Rhizoctonia solani–Infested Soils Challenged by Streptomyces Biocontrol Agents
Source: Front Plant Sci. 2019 Aug 26;10:1038. doi: 10.3389/fpls.2019.01038 (PMC6718142; doi:10.3389/fpls.2019.01038)
Supplement: Supplementary file 1 [file DataSheet_1.zip › Data Sheet 1/Supplement4.pdf]

Supplemental material 4. Most frequent genera found in rhizosphere soils and wheat roots.

	Rhizosphere				Roots	
	Bacteria			Fungi	Bacteria	Fungi
100%	*Aeromicrobium*	*Kribbella*	*Rubrobacter*	*Aspergillus*		
	Arthrobacter	*Modestobacter*	*Skermanella*	*Trichoderma*		
	Balneimonas	*Mycobacterium*	*Sphingomonas*			
	Geodermatophilus	*Nitrospira*	*Steroidobacter*			
	Iamia	*Pseudonocardia*	*Streptomyces*			
	Kaistobacter	*Rhodoplanes*				
>90%	*[Prevotella]*	*Gemmata*	*Planctomyces*	*Cryptococcus*	*Agrobacterium*	*Fusarium*
	Actinoplanes	*Inquilinus*	*Rhizobium*	*Fusarium*	*Pseudomonas*	
	Afifella	*Janthinobacterium*	*Salinibacterium*	*Penicillium*	*Streptomyces*	
	Agrobacterium	*Mesorhizobium*	*Solirubrobacter*	*Podospora*		
	Alicyclobacillus	*Methylibium*	*Sorangium*	*Thanatephorus*		
	Amycolatopsis	*Methylobacterium*	*Sphaerisporangium*			
	Bacillus	*Nocardioides*	*Sporosarcina*			
	Bradyrhizobium	*Nostocoida*	*Streptosporangium*			
	Cellulomonas	*Paenibacillus*	*Virgisporangium*			
	Devosia	*Phenylobacterium*				
	Ellin506	*Pirellula*				
>75%	*A17*	*Fimbriimonas*		*Acremonium*	*Aminobacter*	*Podospora*
	Actinomycetospora	*Flavisolibacter*		*Coniochaeta*	*Bacillus*	
	Agromyces	*Kibdelosporangium*		*Gibberella*	*Caulobacter*	
	Ammoniphilus	*Myxococcus*		*Meliniomyces*	*Devosia*	
	Burkholderia	*Opitutus*		*Minimedusa*	*Janthinobacterium*	
	Cand Koribacter	*Phycicoccus*		*Mortierella*	*Methylobacterium*	
	Cand Xiphinematobacter	*Promicromonospora*		*Oidiodendron*	*Mycobacterium*	
	Craurococcus	*Ramlibacter*		*Olpidium*	*Promicromonospora*	
	Cryocola	*Saccharothrix*		*Rhizophlyctis*	*Rhizobium*	
	DA101	*Sporichthya*		*Scolecobasidium*	*Sphingomonas*	
	Dactylosporangium	*Terracoccus*		*Sordaria*	*Variovorax*	

>50%	*Actinomadura*	*Euzebya*	*Peredibacter*	*Coniosporium*	*[Prevotella]*	*Chitinophaga*	*Salinibacterium*	*Gibberella*
	Aetherobacter	*Gemmatimonas*	*Plesiocystis*	*Cordyceps*	*Achromobacter*	*Cryocola*	*Shinella*	*Rhodotorula*
	Afipia	*Georgenia*	*Pseudomonas*	*Curvularia*	*Actinoplanes*	*Flavobacterium*	*Sphingopyxis*	*Thanatephorus*
	Aminobacter	*Lentzea*	*Rhodobium*	*Dipodascus*	*Aeromicrobium*	*Inquilinus*	*Sporosarcina*	
	Bdellovibrio	*Lysinibacillus*	*Roseomonas*	*Gymnoascus*	*Amycolatopsis*	*Lentzea*	*Stenotrophomonas*	
	Blastococcus	*Massilia*	*Rubellimicrobium*	*Phaeomoniella*	*Arthrobacter*	*Methylopila*		
	Bosea	*Microlunatus*	*Segetibacter*	*Phaeosphaeria*	*Bdellovibrio*	*Microbacterium*		
	Caulobacter	*Mycoplana*	*Variovorax*	*Scytalidium*	*Bosea*	*Mycoplana*		
	Chitinophaga	*Niastella*	*Yonghaparkia*	*Stachybotrys*	*Bradyrhizobium*	*Paenibacillus*		
	Chthoniobacter	*Nonomuraea*			*Burkholderia*	*Pedobacter*		
	Cryptosporangium	*OR-59*			*Cand Xiphinematobacter*	*Phenylobacterium*		
