# Supplementary material for: Decoding Wheat Endosphere–Rhizosphere Microbiomes in Rhizoctonia solani–Infested Soils Challenged by Streptomyces Biocontrol Agents
Source: Front Plant Sci. 2019 Aug 26;10:1038. doi: 10.3389/fpls.2019.01038 (PMC6718142; doi:10.3389/fpls.2019.01038)
Supplement: Supplementary file 1 [file DataSheet_1.zip › Data Sheet 1/Supplement5.pdf]

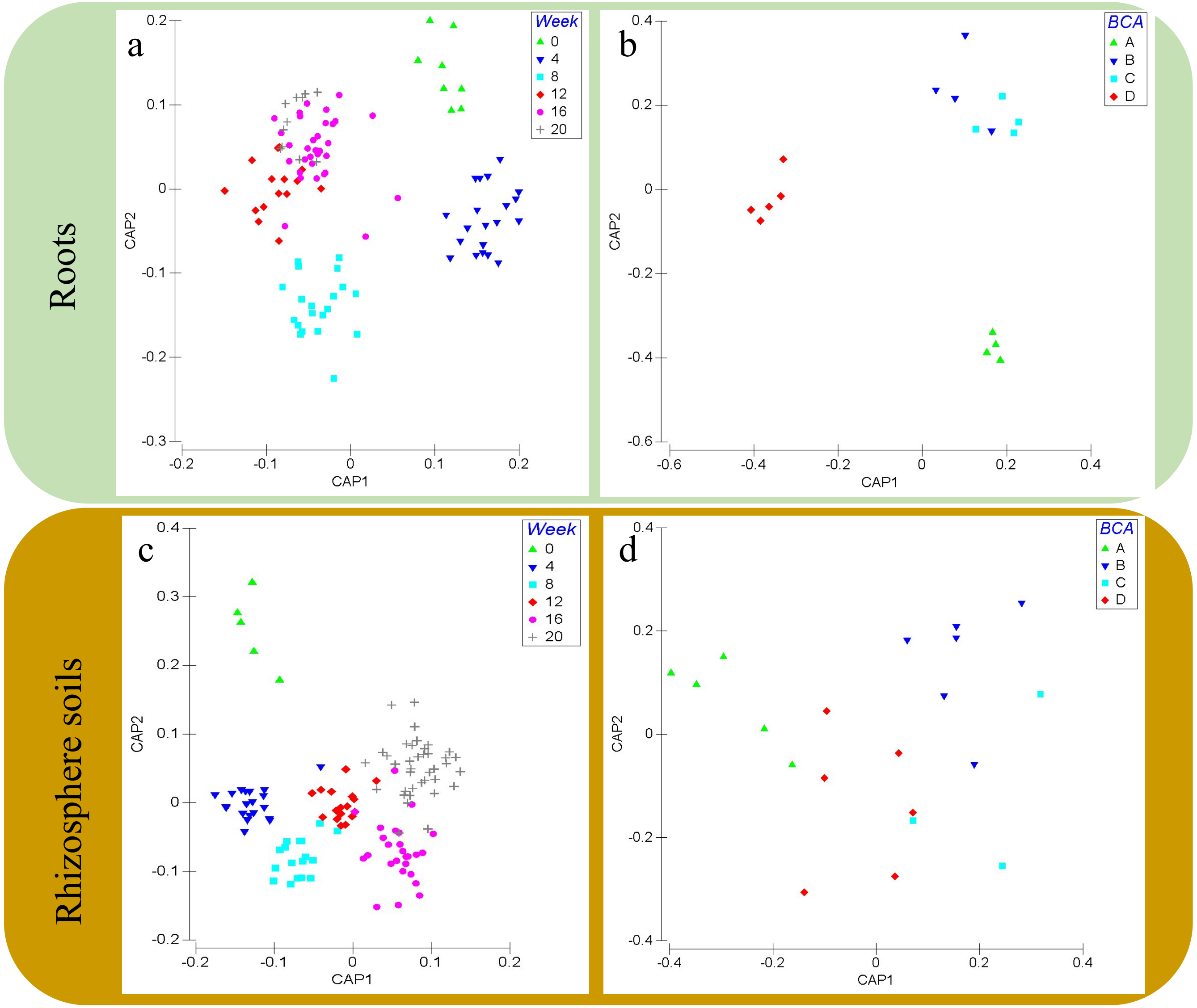

Supplemental information 5. Canonical analysis of principal components (CAP) of seed/root samples (a & b) and rhizosphere soils (c & d) considering the microbiome data; graphs for the 20 weeks period (a & c) and samples collected at week 4 for control (A) versus biocontrol strains F11 (B), EN16 (C) and F5 (D) (b & d). CAP was performed using squared root transformed data, by calculating the resemblance matrix (adding dummy variable), similarity data type and Bray-Curtis similarities.
