# Supplementary material for: Decoding Wheat Endosphere–Rhizosphere Microbiomes in Rhizoctonia solani–Infested Soils Challenged by Streptomyces Biocontrol Agents
Source: Front Plant Sci. 2019 Aug 26;10:1038. doi: 10.3389/fpls.2019.01038 (PMC6718142; doi:10.3389/fpls.2019.01038)
Supplement: Supplementary file 1 [file DataSheet_1.zip › Data Sheet 1/Supplement6.pdf]

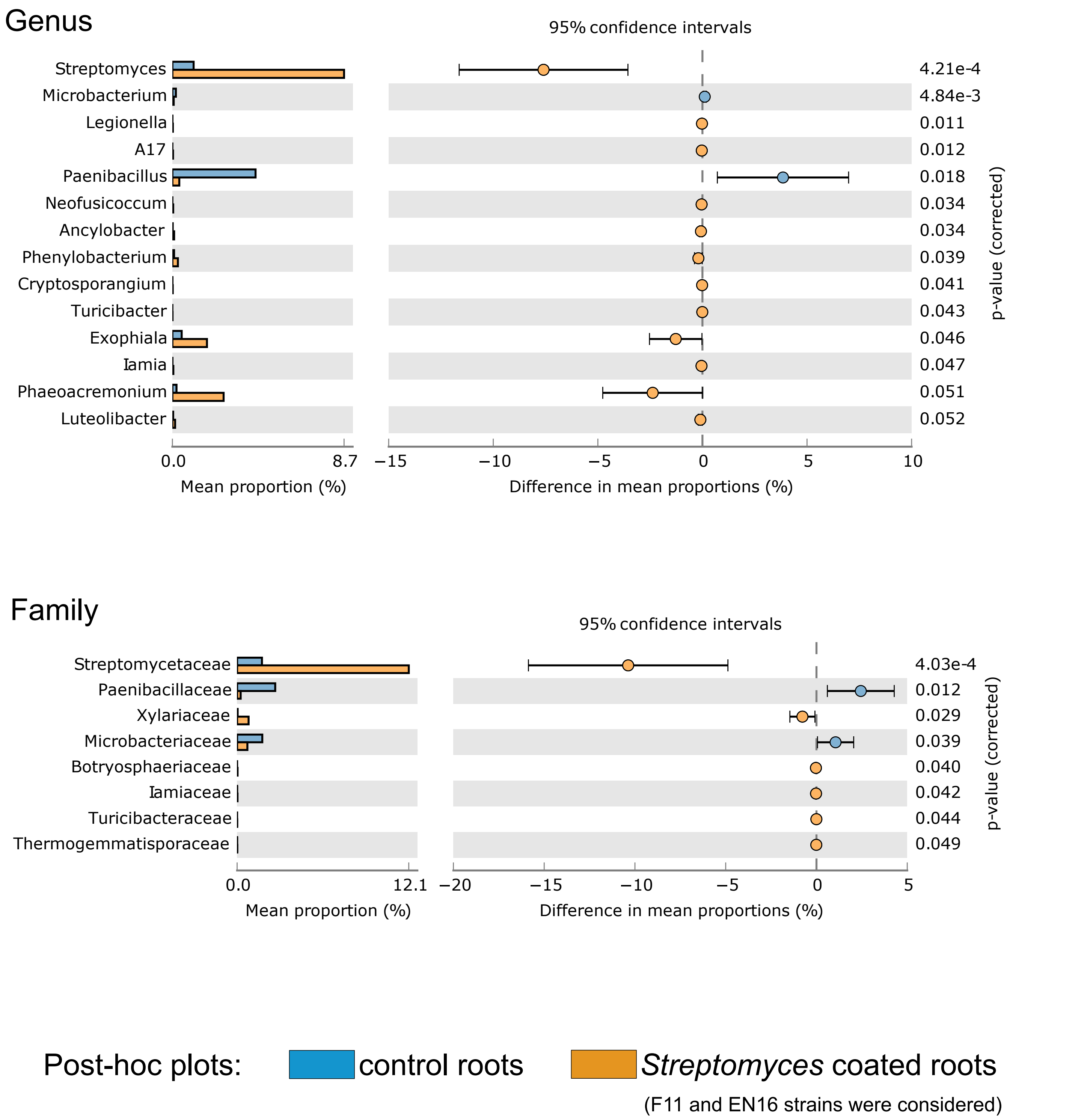

Supplemental information 6. Post-hoc plots of control wheat roots versus *Streptomyces* coated wheat roots at genus and family taxonomic levels (unclassified taxa were removed). Data analyses and statistics were conducted with STAMP; two groups analysis used Welch's t-test (two-sided, Welch's inverted for confidence interval method).
