# Supplementary material for: Decoding Wheat Endosphere–Rhizosphere Microbiomes in Rhizoctonia solani–Infested Soils Challenged by Streptomyces Biocontrol Agents
Source: Front Plant Sci. 2019 Aug 26;10:1038. doi: 10.3389/fpls.2019.01038 (PMC6718142; doi:10.3389/fpls.2019.01038)
Supplement: Supplementary file 1 [file DataSheet_1.zip › Data Sheet 1/Supplement7.pdf]

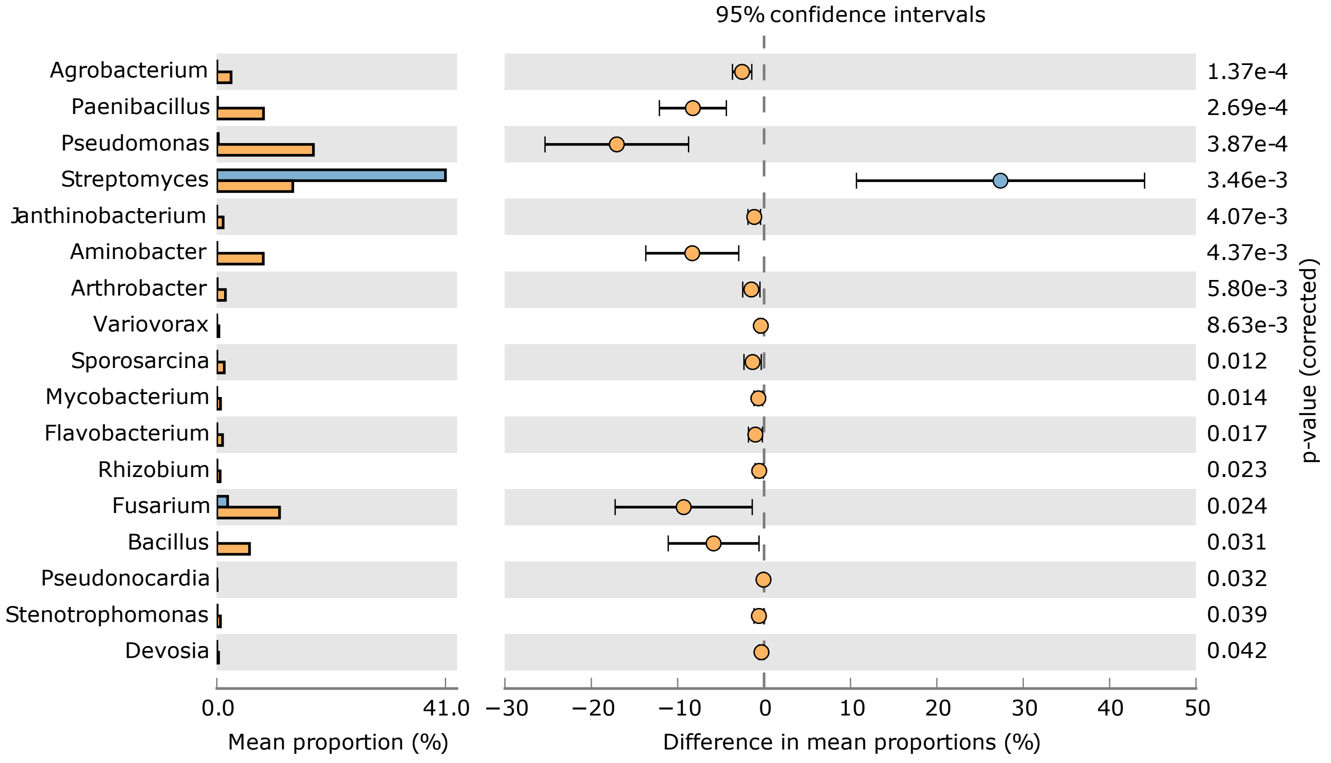

Post-hoc plots: seeds 4 week roots

Supplemental information 7. Post-hoc plots of pairwise wheat seeds/roots for consecutive sampling times at genus level (unclassified taxa were removed); the data for control and biocontrol treatments was pooled for these analyses. Data analyses and statistics were conducted with STAMP; two groups analysis used Welch's t-test (two-sided, Welch's inverted for confidence interval method).

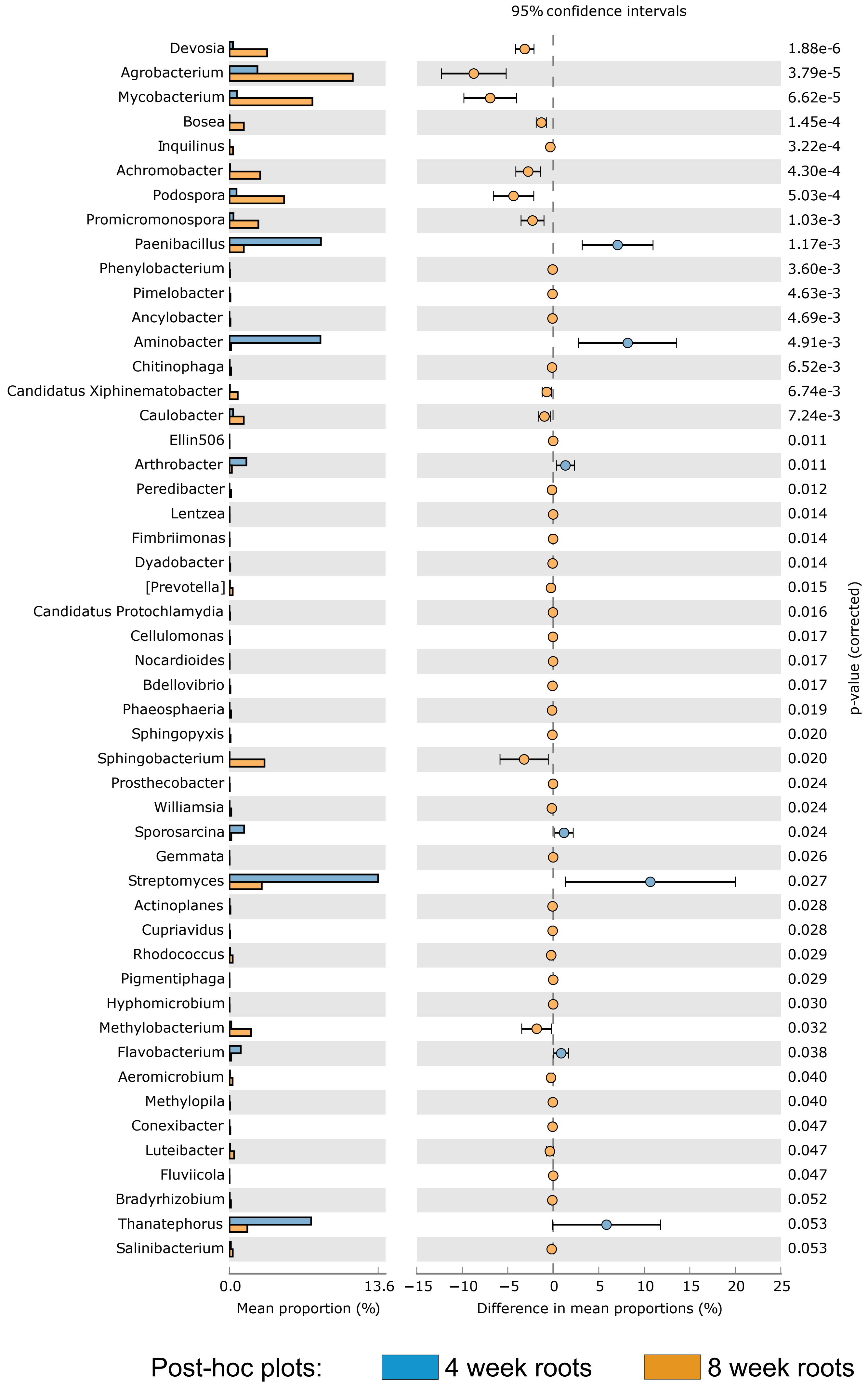

95% confidence intervals
Devosia
Agrobacterium
Mycobacterium
Bosea
Inquilinus
Achromobacter
Podospora
Promicromonospora
Paenibacillus
Phenylobacterium
Pimelobacter
Ancylobacter
Aminobacter
Chitinophaga
Candidatus Xiphinematobacter
Caulobacter
Ellin506
Arthrobacter
Peredibacter
Lentzea
Fimbriimonas
Dyadobacter
[Prevotella]
Candidatus Protochlamydia
Cellulomonas
Nocardioides
Bdellovibrio
Phaeosphaeria
Sphingopyxis
Sphingobacterium
Prosthecobacter
Williamsia
Sporosarcina
Gemmata
Streptomyces
Actinoplanes
Cupriavidus
Rhodococcus
Pigmentiphaga
Hyphomicrobium
Methylobacterium
Flavobacterium
Aeromicrobium
Methylopila
Conexibacter
Luteibacter
Fluviicola
Bradyrhizobium
Thanatephorus
Salinibacterium
1.88e-6
3.79e-5
6.62e-5
1.45e-4
3.22e-4
4.30e-4
5.03e-4
1.03e-3
1.17e-3
3.60e-3
4.63e-3
4.69e-3
4.91e-3
6.52e-3
6.74e-3
7.24e-3
0.011
0.011
0.012
0.014
0.014
0.014
0.015
0.016
0.017
0.017
0.017
0.019
0.020
0.020
0.024
0.024
0.024
0.026
0.027
0.028
0.028
0.029
0.029
0.030
0.032
0.038
0.040
0.040
0.047
0.047
0.047
0.052
0.053
0.053
p-value (corrected)
0.0
13.6
Mean proportion (%)
−15
−10
−5
0
5
10
15
20
25
Difference in mean proportions (%)
Post-hoc plots:
4 week roots
8 week roots

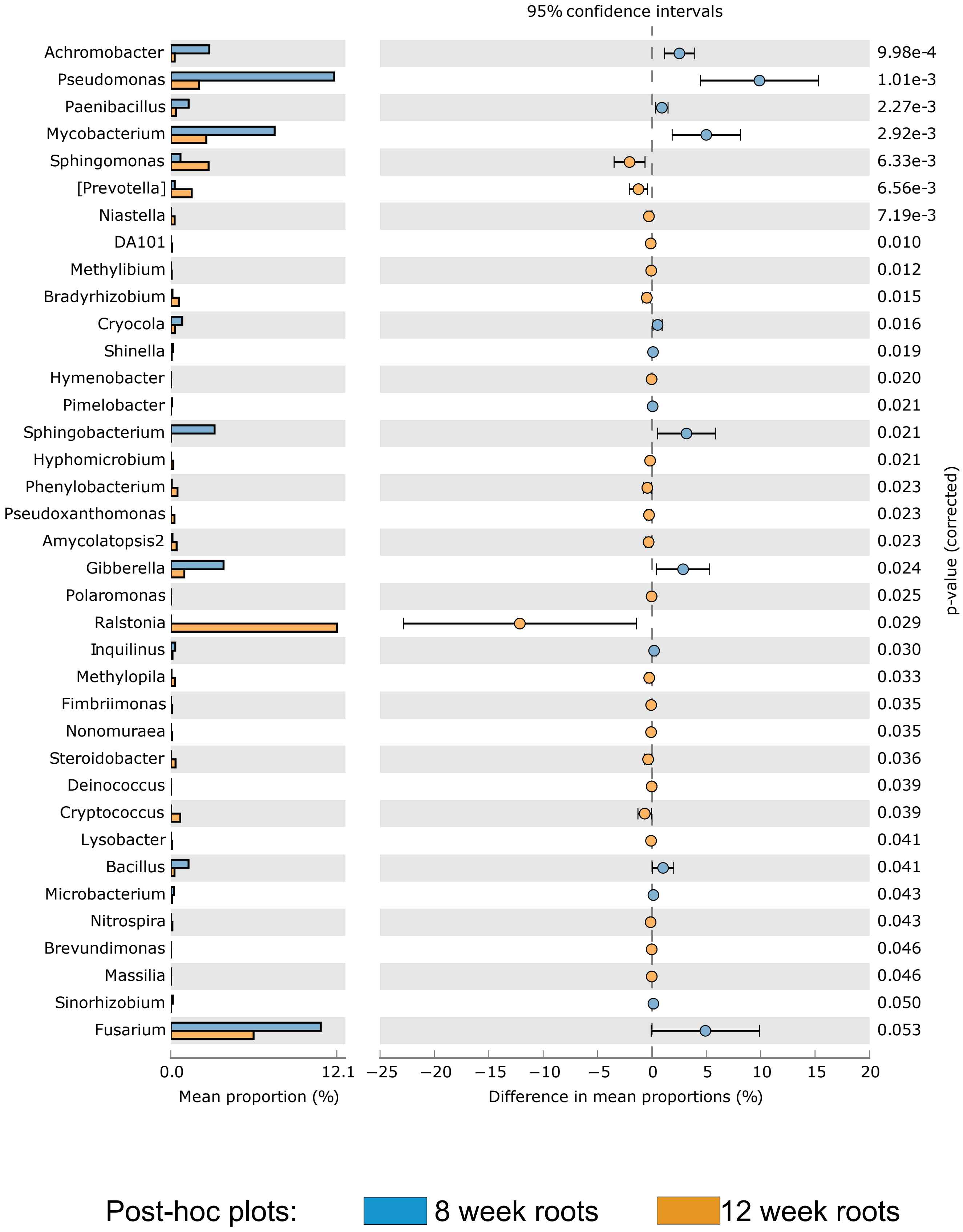
95% confidence intervals
Achromobacter 9.98e-4
Pseudomonas 1.01e-3
Paenibacillus 2.27e-3
Mycobacterium 2.92e-3
Sphingomonas 6.33e-3
[Prevotella] 6.56e-3
Niastella 7.19e-3
DA101 0.010
Methylibium 0.012
Bradyrhizobium 0.015
Cryocola 0.016
Shinella 0.019
Hymenobacter 0.020
Pimelobacter 0.021
Sphingobacterium 0.021
Hyphomicrobium 0.021
Phenylobacterium 0.023
Pseudoxanthomonas 0.023
Amycolatopsis2 0.023
Gibberella 0.024
Polaromonas 0.025
Ralstonia 0.029
Inquilinus 0.030
Methylopila 0.033
Fimbriimonas 0.035
Nonomuraea 0.035
Steroidobacter 0.036
Deinococcus 0.039
Cryptococcus 0.039
Lysobacter 0.041
Bacillus 0.041
Microbacterium 0.043
Nitrospira 0.043
Brevundimonas 0.046
Massilia 0.046
Sinorhizobium 0.050
Fusarium 0.053
0.0
12.1
Mean proportion (%)
−25 −20 −15 −10 −5 0 5 10 15 20
Difference in mean proportions (%)
p-value (corrected)
Post-hoc plots:
8 week roots
12 week roots

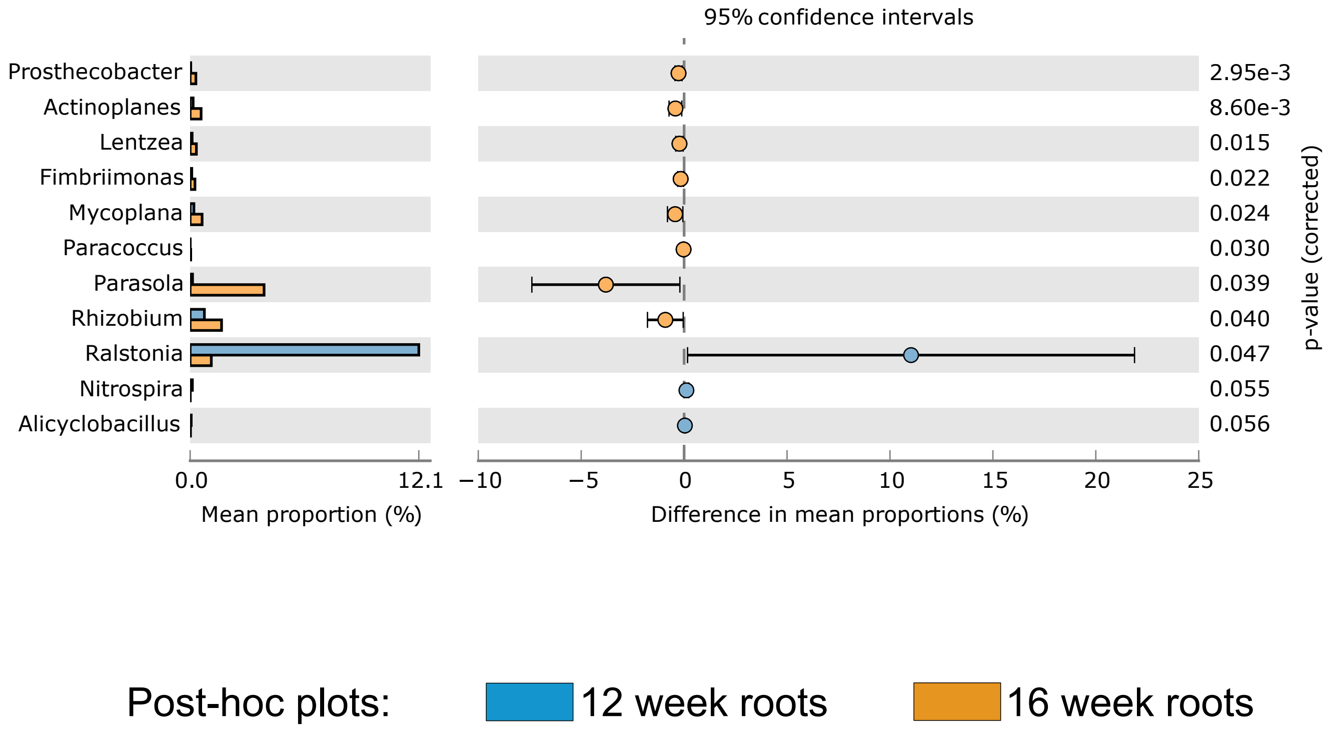
95% confidence intervals
Prosthecobacter
Actinoplanes
Lentzea
Fimbriimonas
Mycoplana
Paracoccus
Parasola
Rhizobium
Ralstonia
Nitrospira
Alicyclobacillus
2.95e-3
8.60e-3
0.015
0.022
0.024
0.030
0.039
0.040
0.047
0.055
0.056
p-value (corrected)
0.0
12.1
Mean proportion (%)
−10
−5
0
5
10
15
20
25
Difference in mean proportions (%)
Post-hoc plots:
12 week roots
16 week roots

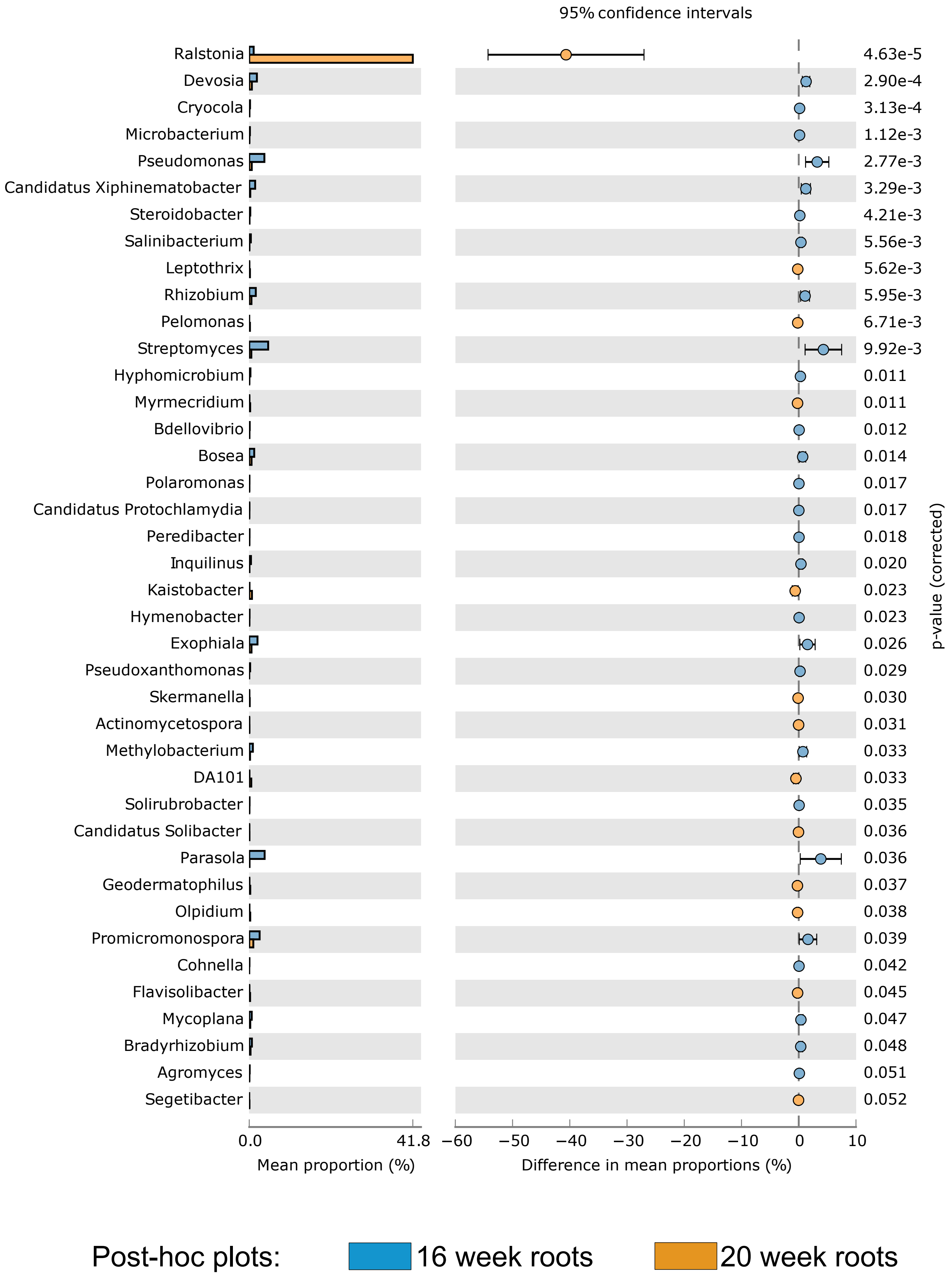

95% confidence intervals
Ralstonia
Devosia
Cryocola
Microbacterium
Pseudomonas
Candidatus Xiphinematobacter
Steroidobacter
Salinibacterium
Leptothrix
Rhizobium
Pelomonas
Streptomyces
Hyphomicrobium
Myrmecridium
Bdellovibrio
Bosea
Polaromonas
Candidatus Protochlamydia
Peredibacter
Inquilinus
Kaistobacter
Hymenobacter
Exophiala
Pseudoxanthomonas
Skermanella
Actinomycetospora
Methylobacterium
DA101
Solirubrobacter
Candidatus Solibacter
Parasola
Geodermatophilus
Olpidium
Promicromonospora
Cohnella
Flavisolibacter
Mycoplana
Bradyrhizobium
Agromyces
Segetibacter
4.63e-5
2.90e-4
3.13e-4
1.12e-3
2.77e-3
3.29e-3
4.21e-3
5.56e-3
5.62e-3
5.95e-3
6.71e-3
9.92e-3
0.011
0.011
0.012
0.014
0.017
0.017
0.018
0.020
0.023
0.023
0.026
0.029
0.030
0.031
0.033
0.033
0.035
0.036
0.036
0.037
0.038
0.039
0.042
0.045
0.047
0.048
0.051
0.052
p-value (corrected)
0.0
41.8
Mean proportion (%)
−60
−50
−40
−30
−20
−10
0
10
Difference in mean proportions (%)
Post-hoc plots:
16 week roots
20 week roots
