# Supplementary material for: Decoding Wheat Endosphere–Rhizosphere Microbiomes in Rhizoctonia solani–Infested Soils Challenged by Streptomyces Biocontrol Agents
Source: Front Plant Sci. 2019 Aug 26;10:1038. doi: 10.3389/fpls.2019.01038 (PMC6718142; doi:10.3389/fpls.2019.01038)
Supplement: Supplementary file 1 [file DataSheet_1.zip › Data Sheet 1/supplement8.pdf]

Rhizosphere soils

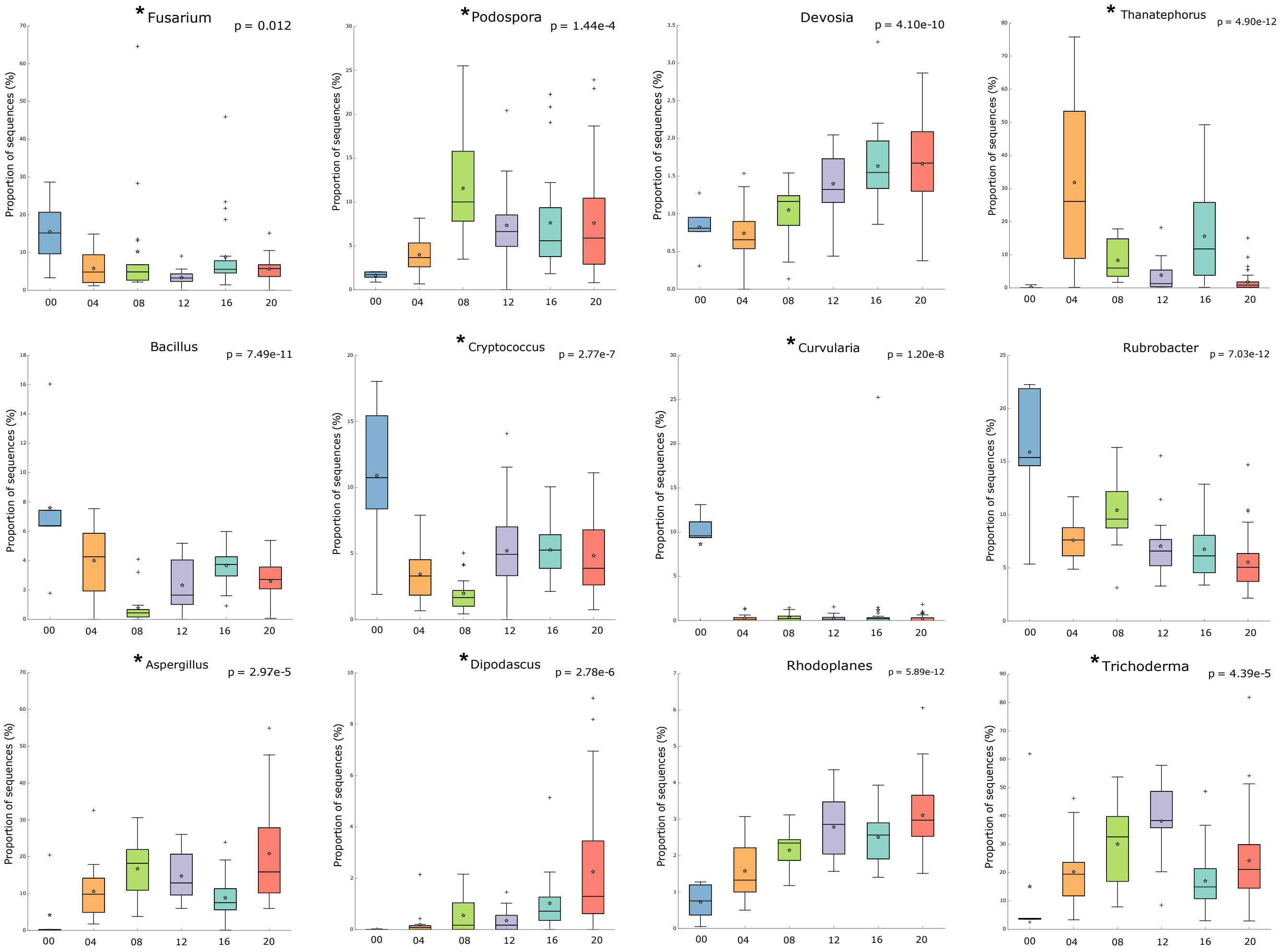

Supplemental information 8. Taxonomic groups affected over time (20 weeks) in rhizosphere soils of wheat crop (* marks the fungal taxa). Data analyses and statistics were conducted with STAMP; Multiple groups' analysis used ANOVA, Tukey-Kramer (0.95) for post-hoc test and Eta-squared for effect size; two groups analysis used Welch's t-test (two-sided, Welch's inverted for confidence interval method).
