# Supplementary material for: Decoding Wheat Endosphere–Rhizosphere Microbiomes in Rhizoctonia solani–Infested Soils Challenged by Streptomyces Biocontrol Agents
Source: Front Plant Sci. 2019 Aug 26;10:1038. doi: 10.3389/fpls.2019.01038 (PMC6718142; doi:10.3389/fpls.2019.01038)
Supplement: Supplementary file 1 [file DataSheet_1.zip › Data Sheet 1/supplement9.pdf]

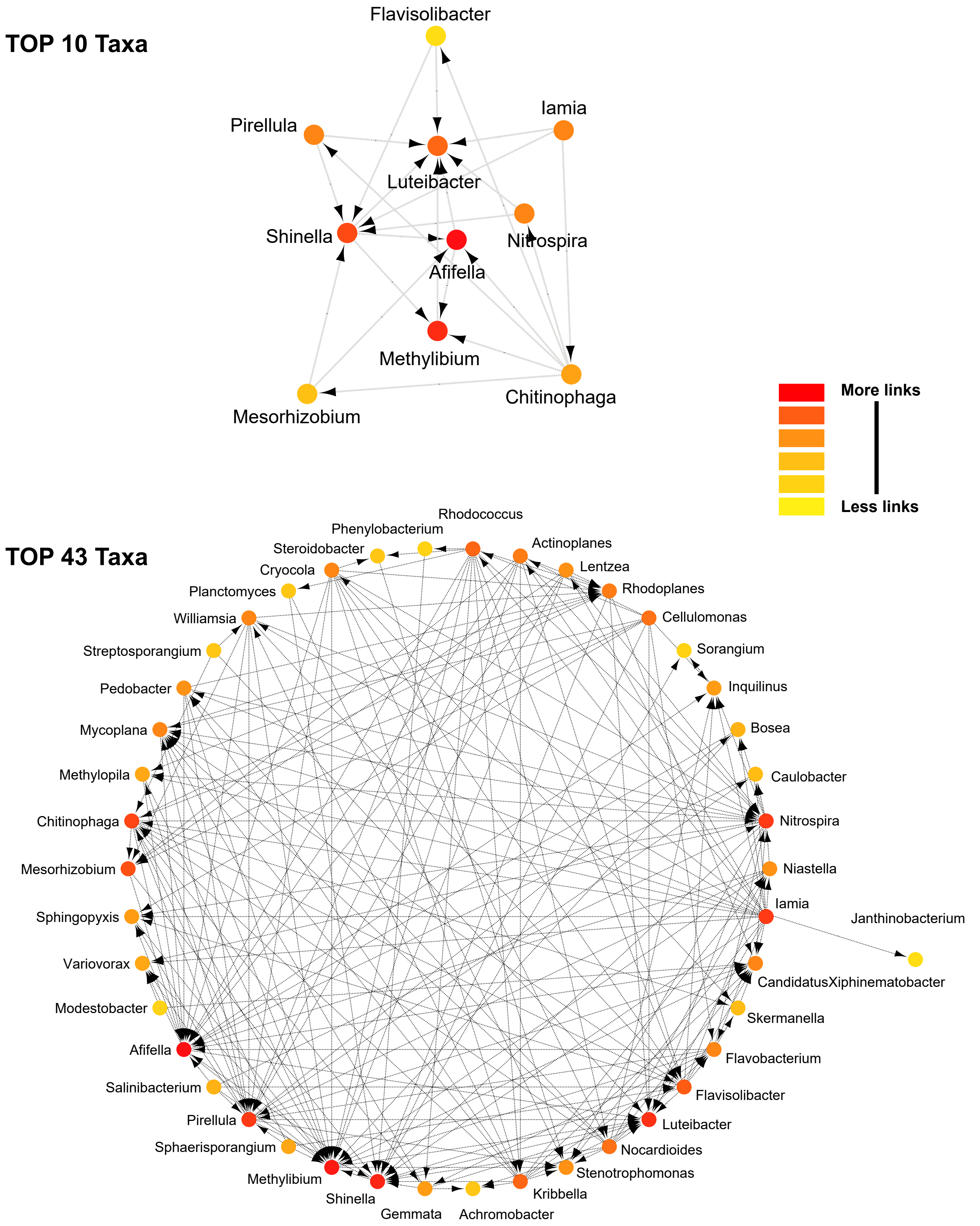

Supplemental information 9. Top 10 and top 43 genera of bacteria (A) found in the centre of the population by network analyses of the community structure (yellow to red: the number of connections is increased). Network analysis was conducted using molecular ecological network analysis pipeline (MENA; http://ieg4.rccc.ou.edu/MENA/) to generate the networks with a cut-off of 0.9, Cytoscape environment to visualize networks and cytoHubba application with maximal clique centrality (MCC) scores to select the top taxa for relevance in roots and rhizosphere soil samples. Bacteria and fungi was analysed in separated files.
